# From formic acid to single-cell protein: genome-scale revealing the metabolic network of *Paracoccus communis* MA5

**DOI:** 10.1186/s40643-022-00544-0

**Published:** 2022-05-18

**Authors:** Sheng Tong, Lizhi Zhao, Daling Zhu, Wuxi Chen, Limei Chen, Demao Li

**Affiliations:** 1 Tianjin Key Laboratory for Industrial Biological Systems and Bioprocessing Engineering, Tianjin Institute of Industrial Biotechnology, Chinese Academy of Sciences, Tianjin, 300308 China; 2 Tianjin Key Laboratory of Brine Chemical Engineering and Resource Eco-Utilization, Tianjin University of Sciences and Technology, Tianjin, 300457 China; 3 National Innovation Centre for Synthetic Biology, Tianjin, 300308 China

**Keywords:** Formic acid, Single-cell protein, Metabolic network, Genome, Transcriptome, *Paracoccus communis*

## Abstract

With the increase in population growth and environmental pollution, the daily protein supply is facing great challenges. Single-cell protein (SCP) produced by microorganism fermentation is a good alternative for substituting plant- and animal-derived proteins. In this study, *Paracoccus communis* MA5 isolated from soil previously demonstrated an excellent ability to synthesize SCP directly from sodium formate. To investigate the central metabolic network of formic acid assimilation and protein synthesis, genome-scale analyses were performed. Genomic analysis showed that complete tetrahydrofolate cycle-, serine cycle-, glycolytic pathway-, tricarboxylic acid (TCA) cycle- and nitrogen metabolism-relevant genes were annotated in the genome. These pathways play key roles in the conversion of formic acid into proteins. Transcriptional analysis showed that sodium formate stress could stimulate the metabolic pathway in response to environmental stress, but weaken the sulfur metabolic pathway to inhibit amino acid synthesis, resulting in a decrease in protein content (30% vs 44%). However, under culture conditions with ammonium sulfate, metabolic pathways associated with protein synthesis were accelerated, causing an increase in protein content (53% vs 44%); while the tetrahydrofolate cycle associated with formic acid assimilation was inhibited, causing a 62.5% decrease in growth rate (OD_600_: 0.21 vs 0.56). These results provide evidence of protein synthesis from sodium formate in strain MA5 at the gene level and lay a theoretical foundation for the optimization of fermentation systems using formic acid as a carbon source.

**Graphical Abstract:**

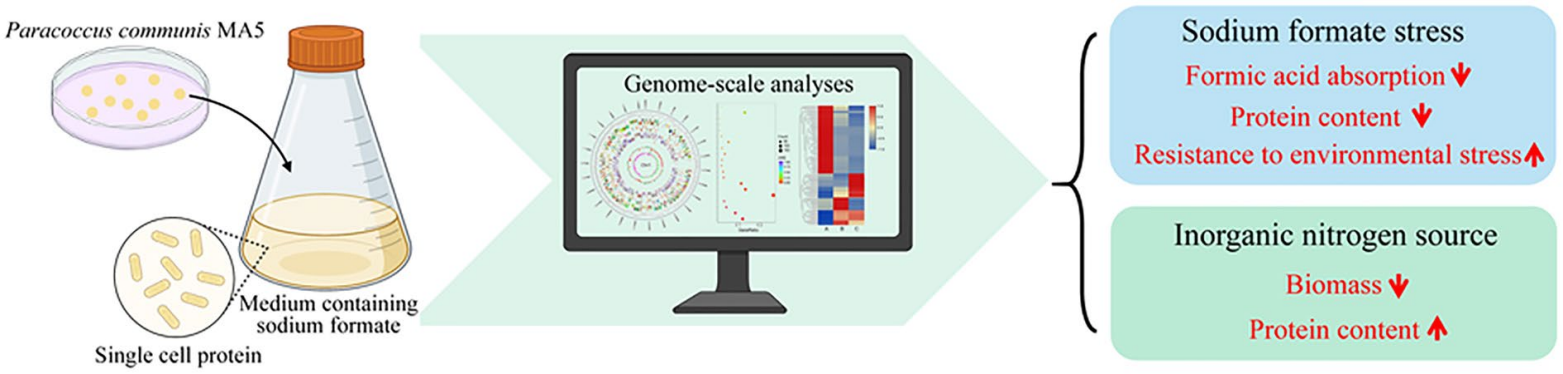

**Supplementary Information:**

The online version contains supplementary material available at 10.1186/s40643-022-00544-0.

## Introduction

Protein is essential for human survival, and its balance is indispensable for human health and well-being. With the development of society, the global demand for protein ingredients has surged due to increased usage of protein. The global protein ingredient market was valued at USD 38 billion in 2019 and is expected to grow at a rate of 9.1% from 2020 to 2027 (Pam et al. 2020), causing enormous pressure on global protein supplies. In addition, accelerating environmental pollution and population growth will lead to insufficient protein supply for traditional agriculture and animal husbandry (Myers et al. 2017; Awange and Kiema 2019; Lv et al. 2021). Therefore, a new production mode is urgently needed to ensure the nutritional value, safety and sustainability of protein.

Single-cell protein (SCP), also known as microbial protein, is produced using agro-industrial byproducts based on the cultivation of protein-producing microorganisms, including bacteria, yeasts, fungi, and microalgae (Ji et al. 2021; Spalvins et al. 2021). SCP consists of 80% protein and contains all essential amino acids (Spalvins et al. 2021; Matassa et al. 2016). Compared to proteins from plants and animals, the production of SCP has many advantages, such as environmental friendliness, water and soil savings, and short production cycles and is not easily affected by the environment (Spalvins et al. 2021). Therefore, single-cell proteins are a good alternative for substituting animal- and plant-derived dietary proteins.

Formic acid, an important organic one-carbon source, is widely used in the industrial manufacturing of leather, pesticides, medicine and rubber. It is found in the secretions of ants and caterpillars in nature (Bennett et al. 1996; Xu et al. 2020). At present, methods for producing formic acid mainly include selective biomass oxidation (Wolfel et al. 2011; Albert et al. 2012, 2014), oxidation of methane and ethylene (Sorokin et al. 2010), hydration of carbon monoxide and carbonylation of methanol with carbon monoxide (Shukla et al. 2005). In addition, the greenhouse gas carbon dioxide which has a wide range of sources, can also be used for producing formic acid by electrochemical system (Agarwal et al. 2011; Kopljar et al. 2014; Wang et al. 2014; Lu et al. 2014; Pletcher 2015; Taheri and Berben 2016; Liu et al. 2018) and hydrogenation (Enthaler 2008; Wang et al. 2015). Therefore, using formic acid as a carbon source to produce protein has the potential to be a sustainable and environmentally friendly technology strategy.

To date, some microorganisms that can utilize formic acid naturally have been reported (Lidstrom 2006; Chistoserdova et al. 2009; Schrader et al. 2009; Bar-Even et al. 2013), and among them, two main metabolic strategies have been found (Bar-Even et al. 2013; Yishai et al. 2017). In the first strategy, formic acid is oxidized to carbon dioxide, and the electrons derived from the oxidation reaction are used to support autotrophic growth through CO_2_ fixation and ATP supply (Bar-Even et al. 2012). In the other strategy, formic acid is assimilated into central carbon metabolism through the tetrahydrofolate cycle and participates in biomass synthesis via assimilation pathways such as the Calvin cycle, the RuBisCo pathway and the serine cycle (Goldberg et al. 1976; Attwood and Harder 1978; Jansen et al. 1984; Vuilleumier et al. 2009). However, these natural formate-utilizing microorganisms generally grow slowly and metabolize formic acid inefficiently (Xu et al. 2020). Therefore, a detailed and comprehensive regulatory network of formic acid metabolism urgently needs to be explored to provide guidance for the optimization and modification of formic acid metabolism. In recent years, formic acid has been biologically utilized to produce organic compounds such as ethanol and acetic acid (Kim et al. 2019; Xu et al. 2020); however, single-cell protein synthesis from formic acid has yet to be reported. The strain showing high homology with *P. communis* G9-1 was isolated from soil and named *P. communis* MA5 (Zhao et al. 2021). Previous studies have shown that strain MA5 could produce SCP directly from sodium formate. When cultured with different nitrogen sources or sodium formate concentrations, the growth rate and protein content were significantly changed. To achieve efficient utilization of formic acid and high production of SCP, comprehensive metabolic pattern analyses of strain MA5 were carried out at the genomic and transcriptomic levels.

## Materials and methods

### Strain and culture conditions

*P. communis* strain MA5 was isolated by our group and deposited at the CGMCC with accession number CGMCC No.21106. Strain MA5 was routinely grown in LB broth (10 g/L tryptone, 5 g/L yeast extract and 10 g/L NaCl) overnight at 37 ℃ and then spread onto a screening plate containing sodium formate (8 g/L HCOONa, 0.5 g/L NaCl, 0.5 g/L MgCl_2_.6H_2_O, 0.1 g/L CaCl_2_.2H_2_O, 0.5 g/L KCl, 0.2 g/L KH_2_PO_4_, 1 g/L yeast powder and 15 g/L agar) at 37 ℃ to maintain bacterial activity. For transcriptome sequencing, bacteria were washed from LB plates with sterile saline solution and inoculated at 1% (v/v) into three different fermentation media at 37 ℃ for 48 h. These treatments were named Group A (8 g/L HCOONa, 1 g/L yeast powder), Group B (20 g/L HCOONa, 1 g/L yeast powder) and Group C (8 g/L HCOONa, 2 g/L (NH_4_)_2_SO_4_), and the other components were consistent with the screening medium above. Each group was analyzed in three independent biological samples. The bacteria grown in Group A were also selected for genome sequencing.

### Analysis of cell growth

Cell growth was indirectly observed by measuring the OD_600_. After centrifugation at 10,000 g for 10 min, the cells cultured at 37 ℃ for 48 h in fermentation media were washed in sterile water twice and diluted to a suitable concentration. Then, 200 μL of cell suspensions were added to a 96-well plate, and the OD_600_ values were measured by a microplate reader (BioTek, Neo2). The experiments were performed in triplicate on three biological replicates, and error bars ( ±) represent the standard deviation of three replicates.

### Determination of protein content

Determination of the total protein content was performed as described (Chen et al. 2016) with some modifications. Briefly, the bacteria cultured at 37 ℃ for 48 h in fermentation media were washed with PBS 3 times and then resuspended in 10 mL of distilled water. After ultrasonication for 10 min, 1 mL of the above mentioned suspension was mixed with 5 mL of NaOH (0.1 M), and the final sample was obtained by boiling for 10 min. The OD_595_ was measured using the Bradford method by a microplate reader (BioTek, Neo2), and the protein concentration was calculated based on a bovine serum albumin standard curve. The experiments were performed in triplicate on three biological replicates, and error bars ( ±) represent the standard deviation of three replicates.

### Determination of formic acid content

The formic acid content was determined using high-performance liquid chromatography as previously described (Morris 1987). Briefly, the sample was diluted with 10 mM sulfuric acid to a suitable concentration (within the range of the standard curve). Then, formic acid content was measured by an Aminex HPX-87H column (Bio-Rad, USA) with 5 mM sulfuric acid as the mobile phase at a 0.5 mL/min flow rate and a 10 μL injection volume. Chromatographic pure formic acid was used to prepare the standard curve, and the formic acid content in the sample was calculated (g/L). The experiments were performed in triplicate on three biological replicates, and error bars ( ±) represent the standard deviation of three replicates.

### Genome sequencing and analysis

Genomic DNA was isolated from strain MA5 using a Bacterial Genome Extraction Kit according to the manufacturer’s instructions (Tiangen, China). DNA integrity was detected by agarose gel electrophoresis (1% w/v), and the concentration was measured in a NanoDrop 2000 (Thermo, USA). The genome of strain MA5 was sequenced by the third-generation sequencer PacBio. The SMRT Bell™ Template Kit was used to construct a 10K SMRT Bell library, and the size of the inserted fragment was detected using an Agilent 2100 BioAnalyzer. Library construction and sequencing were performed at Beijing Novogene Bioinformatics Technology Co., Ltd. After removing low-quality sequences from raw data, the high-quality clean data that could be used for analysis were assembled using SMRT Link v5.0.1 software. The coding sequences were predicted using GeneMarkS software, and their functions were annotated through comparisons with the NR, COG and KEGG databases. The genome sequenced has been deposited at GenBank under accession number CP087597-CP087599.

### Transcriptome sequencing and analysis

Total RNA was extracted from strain MA5 using the RNAprep pure Cell/Bacteria Kit according to the instructions (Tiangen, China). RNA integrity and concentrations were detected by agarose gel electrophoresis (1% w/v) and an Agilent 2100 BioAnalyzer. Transcriptomic libraries were constructed according to the manufacturer’s instructions, and 2 × 150-bp paired-end sequencing was performed on an Illumina platform at Beijing Novogene Bioinformatics Technology Co., Ltd. The filtered reads were mapped to the annotated genome of strain MA5 using Bowtie2 (Langmead and Salzberg 2012). BAM files were then converted from alignment files using SAMtools v1.3.1 (Li and Durbin 2009). Read counts per gene were collected using HTSeq v0.6.1 (Anders et al. 2015), followed by exploration of the differential expression across pairwise comparisons of the three conditions using DESeq v1.26.0 (Anders and Huber 2010). KEGG enrichment analysis of differentially expressed genes (p value < 0.05) was performed in ClusterProfiler software (Morin et al. 2018), and a heatmap was drawn using TBtools (Chen et al. 2020).

### Gene expression level detection by real-time polymerase chain reaction (PCR)

Total RNA was isolated as described above and converted to cDNA using a FastKing RT Kit (Tiangen, China). The expression of target genes was analyzed by real-time PCR with *dnaN* as the reference gene, and the PCR procedure was set according to the instructions of the PowerUp SYBR Green Master Mix Kit (Thermo, USA). The primers used in this study are listed in Additional file 1: Table S1.

## Results

### Physiological parameter detection of *P. communis* MA5

To investigate the characteristics of formic acid metabolism and protein synthesis of strain MA5 in sodium formate stress or different nitrogen sources (organic or inorganic nitrogen), formic acid consumption, biomass and protein content were monitored under selected culture conditions. Compared to the standard culture condition (A: normal sodium formate concentration and organic nitrogen), formic acid consumption and protein content were decreased significantly by 67% and 31.8% in the sodium formate stress treatment (B: sodium formate stress and organic nitrogen), while a 62.5% decrease in growth rate and a 20.4% increase in protein content were observed under the culture conditions with ammonium sulfate instead of yeast extract (C: normal sodium formate concentration and inorganic nitrogen) (Table 1).

**Table 1:** Physiological parameter detection of strain MA5 under different culture conditions

Sample	Formic acid absorption (g/L)	Biomass (OD_600_)	Protein content (%)
A	4.61 ± 0.24	0.56 ± 0.07	44 ± 1.55
B	1.51 ± 0.22 ^a^	0.49 ± 0.09^b^	30 ± 1.79^a^
C	4.40 ± 0.37 ^b^	0.21 ± 0.05^a^	53 ± 1.31^a^

Culture condition A: 8 g/L HCOONa, 1 g/L yeast powder, 0.5 g/L NaCl, 0.5 g/L MgCl_2_.6H_2_O, 0.1 g/L CaCl_2_.2H_2_O, 0.5 g/L KCl, 0.2 g/L KH_2_PO_4_. Culture condition B: 20 g/L HCOONa, 1 g/L yeast powder, 0.5 g/L NaCl, 0.5 g/L MgCl_2_.6H_2_O, 0.1 g/L CaCl_2_.2H_2_O, 0.5 g/L KCl, 0.2 g/L KH_2_PO_4_. Culture condition C: 8 g/L HCOONa, 2 g/L (NH4)_2_SO_4_, 0.5 g/L NaCl, 0.5 g/L MgCl_2_.6H_2_O, 0.1 g/L CaCl_2_.2H_2_O, 0.5 g/L KCl, 0.2 g/L KH_2_PO_4_. Error bars ( ±) represent the standard deviation of three replicates. Letters indicate statistically significant differences, as determined by Student’s *t* test (^a^indicates p < 0.01; ^b^indicates no significance)

### Extraction and quality analysis of genomic DNA and total RNA from *P. communis* MA5

For omics analysis, the genomic DNA and total RNA of *P. communis* MA5 were first extracted and their quality were evaluated. The electrophoresis results revealed that the main bands of genomic DNA and RNA showed almost no degradation (Additional file 1: Figure S1), and RNA integrity was further confirmed by RNA integrity number (RIN) detection (Additional file 1: Table S2, RIN > 8). Moreover, the concentrations of RNA (> 300 ng/μL) and genomic DNA (84.2 ng/μL) both reached the requirements of library construction (Additional file 1: Table S2 and S3). These results suggest that the above samples can be used for whole-genome and transcriptome sequencing.

### Genome assembly, gene prediction and functional annotation

The draft genome of strain MA5 consists of 3 contigs, including two annular chromosomes and an annular plasmid (Additional file 1: Figure S2). The genome size is approximately 4,473,736 bp with a GC content of 68.56%, and the lengths of the 3 contigs are 1,412,597 bp (Chr1), 2,389,614 bp (Chr2) and 671,525 bp (Plas1). A total of 4361 genes comprising 4,013,268 bp were predicted, 4301 of which were protein-coding genes, and 60 were RNA genes. In addition, five prophages with sizes from 7164 bp to 44,730 bp and a CIRSPR region together with cas3 upstream of this region were identified in the genome of MA5 (Table 2).

**Table 2:** Genome statistics

Attribute	Value	% of total*
Genome size (bp)	4,473,736	100
Chr1 (bp)	1,412,597	31.58
Chr2 (bp)	2,389,614	53.41
Plas1 (bp)	671,525	15.01
GC content (bp)	3,067,193	68.56
Coding region (bp)	4,013,268	89.71
Coding genes (No.)	4361	
Protein-coding genes (No.)	4301	98.62
RNA genes (No.)	60	1.38
Prophages (No.)	5	
CIRSPR region (No.)	1	

^*^The total is based on either the size of the genome in base pairs or the total number of protein-coding genes in the annotated genome

**Fig. 1:**
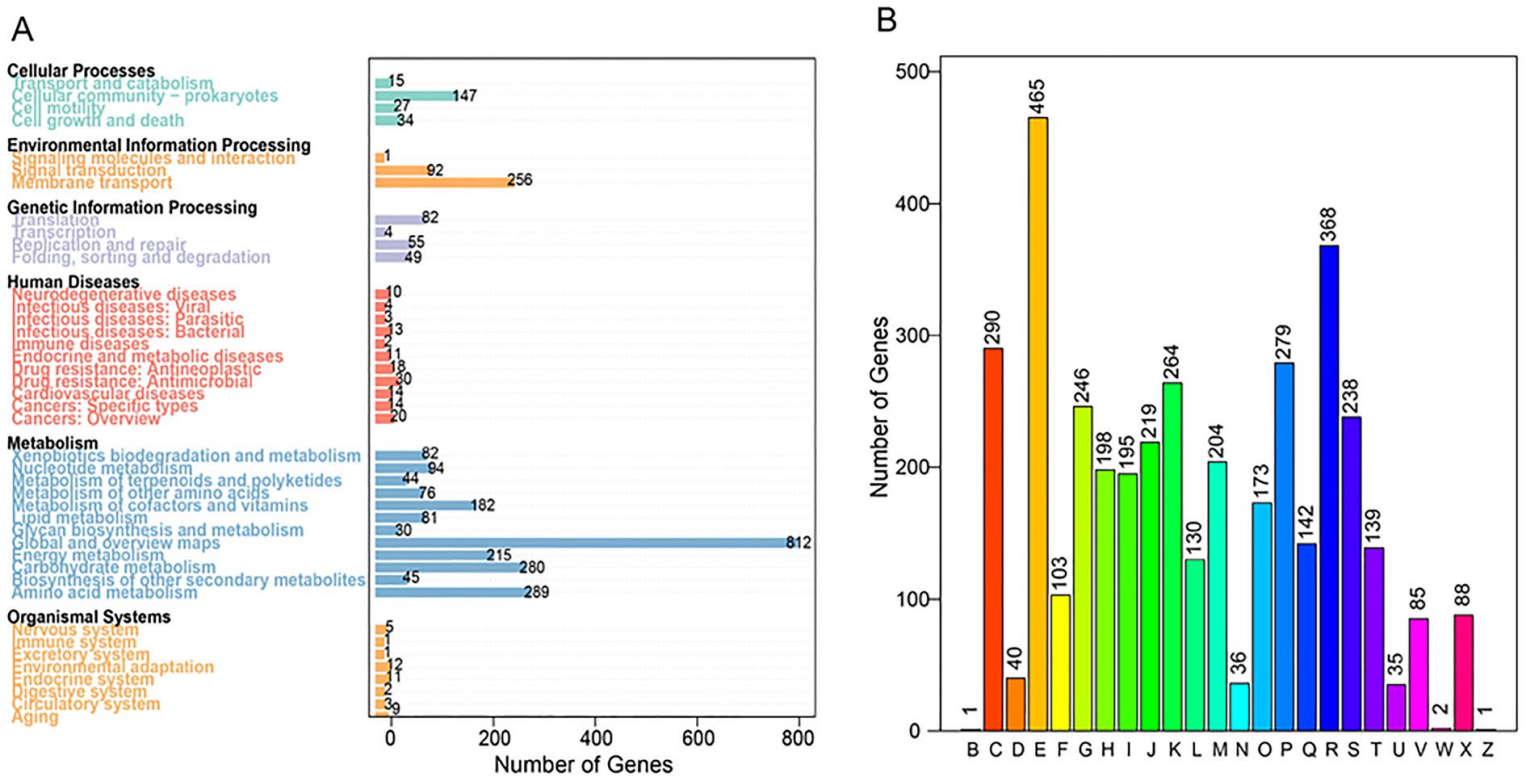
KEGG pathway mapping (**A**) and COG functional assignment (**B**) of genes in the genome of strain MA5. The numerical value above the column represents the number of genes in the corresponding pathway or classification. The genes were mainly concentrated in the category of metabolism in KEGG pathway mapping and on amino acid transport and metabolism in COG function assignment

According to the KO assignment and KEGG pathway mapping, 2355 (54.75%) protein-coding genes could be assigned to 222 metabolic pathways (one gene may correspond to many pathways). These pathways are classified into 6 categories: cellular processes, environmental information processing, genetic information processing, human diseases, metabolism, and organismal systems. The vast majority of genes (2230) were concentrated in the category of metabolism, and among them, 13% of genes (289/2230) were focused on amino acid metabolism (the 2nd most in metabolism) (Fig. 1A). Similarly, using COG function assignment, 3477 (80.84%) protein-coding genes could be classified into 24 COG categories, and the most abundant category of genes (465) was related to amino acid transport and metabolism (Fig. 1B and Additional file 1: Table S4). Based on the results above, we suspect that active amino acid metabolic pathways in strain MA5 can explain the efficient synthesis of protein.

### Genome-scale reconstruction of the central metabolic networks in strain MA5

**Fig. 2:**
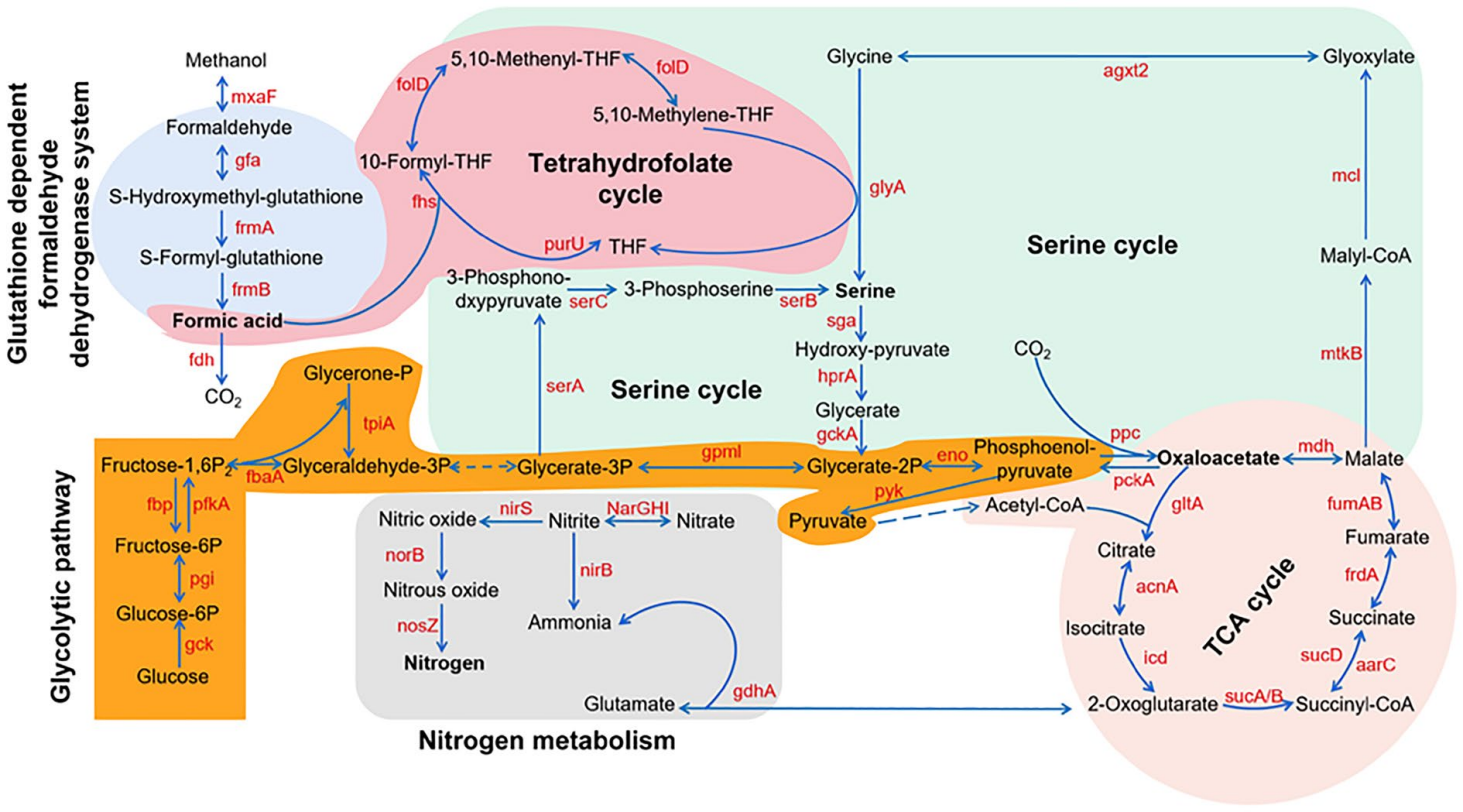
Proposed central metabolism pathways of strain MA5. *maxF* methanol dehydrogenase, *gfa* S-(hydroxymethyl)glutathione synthase, *frmA* S-(hydroxymethyl)glutathione dehydrogenase, *frmB* S-formylglutathione hydrolase, *fdh* formate dehydrogenase, *fhs* formate-tetrahydrofolate ligase, *purU* formyltetrahydrofolate deformylase, *folD* methylene-tetrahydrofolate dehydrogenase/methenyltetrahydrofolate cyclohydrolase, *glyA* glycine hydroxymethyltransferase, *sga* serine glyoxylate transaminase, *hprA* glycerate dehydrogenase, *gckA* glycerate 2-kinase, *gpmI* 2,3-bisphosphoglycerate-independent phosphoglycerate mutase, *serA* D-3-phosphoglycerate dehydrogenase, *serC* phosphoserine aminotransferase’, *serB* phosphoserine phosphatase, *eno* enolase, *ppc* phosphoenolpyruvate carboxylase, *pckA* phosphoenolpyruvate carboxykinase, *mdh* malate dehydrogenase, *mtkB* malate-CoA ligase, *mcl* malyl-CoA/(S)-citramalyl-CoA lyase, *agxt2* alanine-glyoxylate transaminase, *tpiA* triosephosphate isomerase, *fbaA* fructose-bisphosphate aldolase, *pfkA* 6-phosphofructokinase, *fbp* fructose-1,6-bisphosphatase, *pgi* glucose-6-phosphate isomerase, *gck* glucokinase, *pyk* pyruvate kinase, *gltA* citrate synthase, *acnA* aconitate hydratase, *icd* isocitrate dehydrogenase, *sucA* 2-oxoglutarate dehydrogenase E1 component, *sucB* 2-oxoglutarate dehydrogenase E2 component, *sucD* succinyl-CoA synthetase alpha subunit, *aarC* succinyl-CoA:acetate CoA-transferase, *frdA* fumarate reductase flavoprotein subunit, *fumAB* fumarate hydratase, *narGHI* nitrate reductase, *nirBS* nitrite reductase, *norB* nitric oxide reductase, *nosZ* nitrous oxide reductase, *gdhA* glutamate dehydrogenase. Dotted arrows indicate multistep reactions

The central metabolic networks of strain MA5 were proposed according to the genome analysis results and included an integrated glutathione-dependent formaldehyde dehydrogenase system, tetrahydrofolate cycle, serine cycle, glycolytic pathway, TCA cycle and nitrogen metabolism (Fig. 2). In strain MA5, formic acid is transformed to methylene-tetrahydrofolate through tetrahydrofolate ligase, methylene-tetrahydrofolate dehydrogenase and methylene-tetrahydrofolate hydrolase. The ligase is encoded by the *fhs* gene, and the dehydrogenase and hydrolase are encoded by the same gene, named *folD*. Thereafter, methylene-tetrahydrofolate reacts with glycine via glycine hydroxymethyltransferase (glyA) to produce l-serine, entering biomass synthesis metabolism through the serine cycle. The serine cycle is the only way to assimilate formic acid into central carbon metabolism and consists of two parts. One is the serine regeneration cycle, and the other is the glyoxylic acid regeneration cycle. The common branch of both is composed of serine glyoxylate aminotransferase encoded by the *sga* gene, hydroxypyruvate reductase encoded by the *hpr* gene, and glycerate kinase encoded by the *gck* gene. Moreover, a phosphoenolpyruvate carboxylase was found in MA5. The enzyme can catalyze the carboxylation of carbon dioxide with phosphoenolpyruvate to produce oxaloacetic acid (an important intermediate in the TCA cycle), which is able to neutralize carbon dioxide (Puri et al. 2020). The common glycolytic pathway and TCA cycle were also activated in MA5. Through these pathways, formic acid is drastically introduced into the central metabolism.

For nitrogen metabolism, there are two branches in MA5. One is the dissimilative nitrate reduction pathway catalyzed by nitrate reductase and nitrite reductase. In this pathway, nitrate is first reduced to ammonia, and then ammonia is transformed to glutamate via glutamate dehydrogenase and subsequently enters glutamate metabolism. The other branch is the denitrification pathway catalyzed by nitrate reductase, nitrite reductase, and nitric oxide reductase, in which nitrate is finally reduced to nitrogen and discharged from the body. With the help of the entire central metabolic network, protein synthesis based on formic acid as a carbon source proceeds smoothly.

### RNA-seq and differential expression analysis

**Fig. 3:**
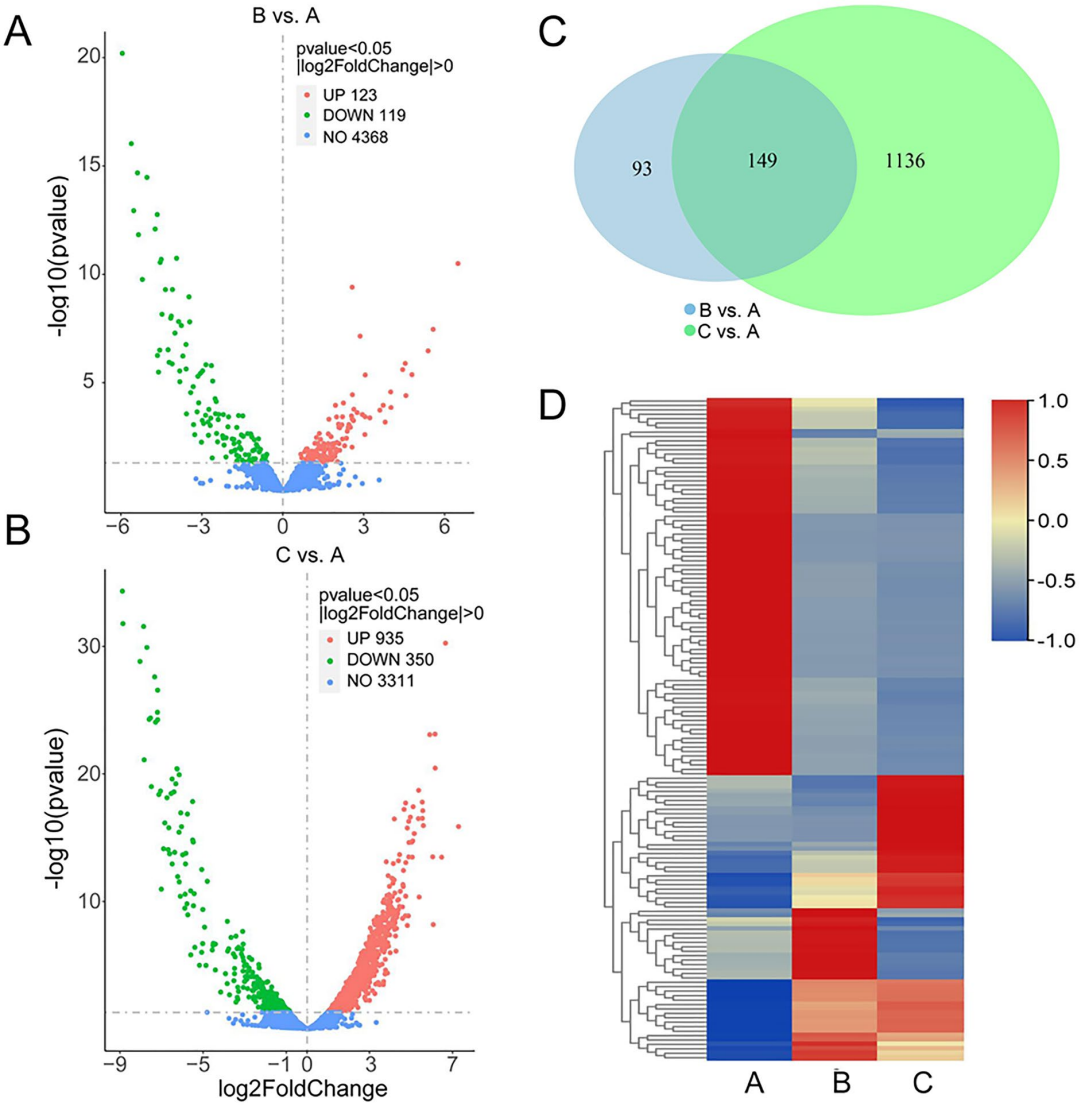
Analysis of differentially expressed genes (DEGs) under three conditions. **A** Volcano plot of DEGs in the B vs. A group. **B** Volcano plot of DEGs in the C vs. A group. Red dots represent upregulated DEGs, green dots represent downregulated DEGs, and blue dots represent unchanged genes. The DEGs were identified with a p value < 0.05 and |log2FoldChang|> 0. **C** Venn diagram between the B vs. A group and the C vs. A group. **D** Heatmap of shared genes involved in both groups. The heatmap was drawn using TBtools software, and the rows were normalized and clustered

Based on the experimental results, gene expression profiling of bacteria grown in different media was performed via RNA-seq analyses as detailed in the Materials and methods section. A total of 1.55, 1.51, and 1.53 million reads were generated, and 99.3%, 99.4%, and 98.9% were mapped to the strain MA5 genome in the A-C sample, respectively. A comparison of differentially expressed genes (DEGs) between B (or C) and A was performed. The analysis results revealed a total of 123 transcripts upregulated and 119 downregulated in the B vs. A group (Fig. 3A), and 935 transcripts were upregulated and 350 were downregulated in the C vs. A group (Fig. 3B). Cross-comparison between the B vs. A group and the C vs. A group showed 149 DEGs shared within both groups (Fig. 3C). Among them, more than half of the genes were downregulated compared with the control treatment (Fig. 3D), and the specific DEG annotations are listed in Additional file 1: Table S5.

### Sodium formate stress results in elevated expression of peptidoglycan synthesis-related genes but a weakening in the sulfur metabolic pathway

**Fig. 4:**
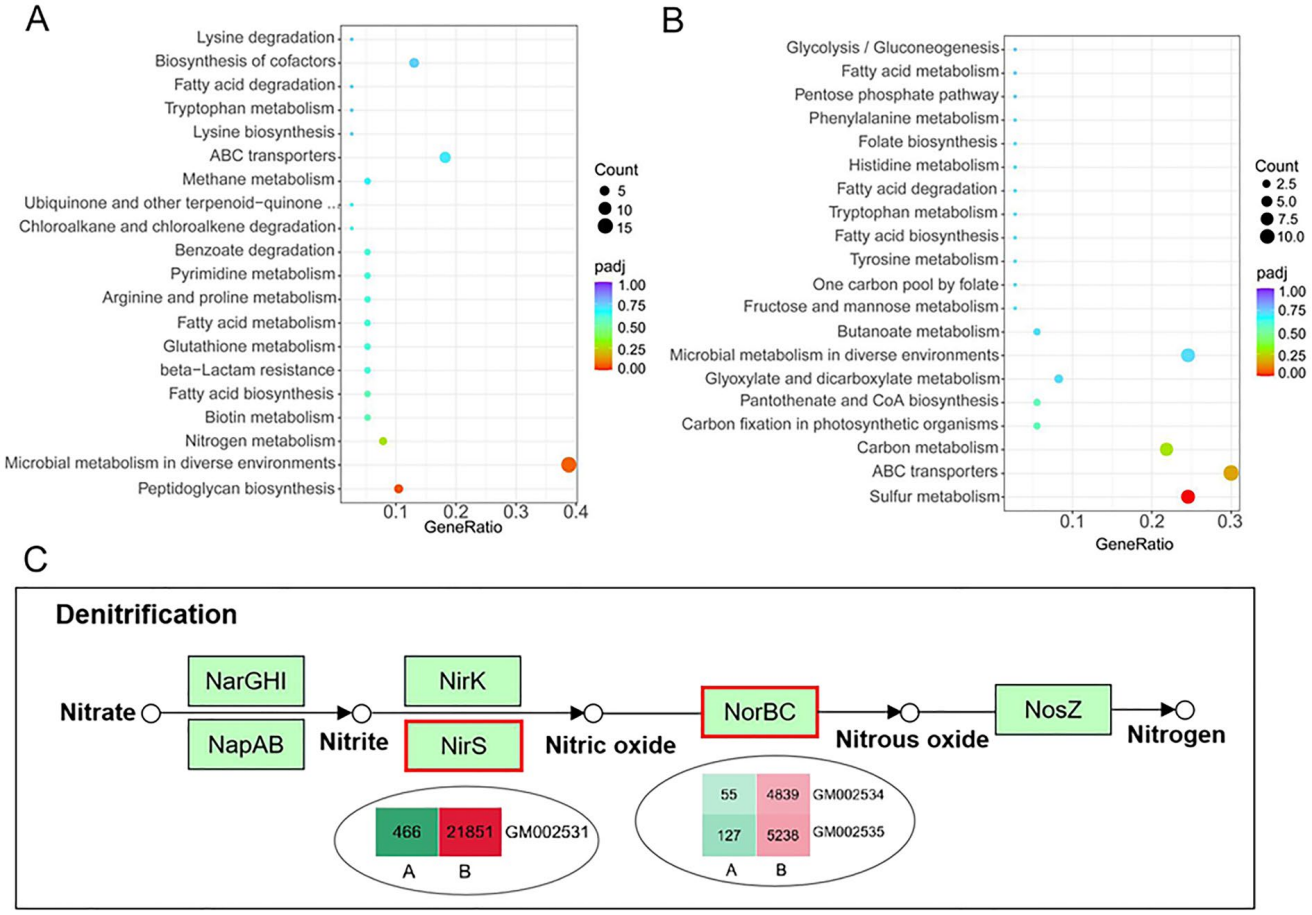
KEGG pathway enrichment of DEGs in the B vs. A group. **A** KEGG pathway enrichment of the upregulated DEGs. **B** KEGG pathway enrichment of the downregulated DEGs. The size of the circle represents the number of DEGs in the pathway, and the color of the circle represents the padj value (p value adjusted to false discovery rate), reflecting the enrichment degree. **C** The DEGs involved in the denitrification pathway. The genes encoding enzymes in the rectangular box with a red border were significantly upregulated, and the FPKM of each gene is shown in the rectangle inside the ellipse

KEGG pathway enrichment network analyses revealed that the upregulated DEGs of the B vs. A group showed significant enrichment in the peptidoglycan synthesis pathway containing ligase, N-acetylglucosamine transferase, and peptidoglycan glycosyltransferase (Fig. 4A). Peptidoglycan is the main component of the bacterial cell wall, offering the function of increasing resistance to environmental stress. Therefore, the upregulated expression of peptidoglycan synthesis-related genes under sodium formate stress is reasonable. Similarly, sharply grouped genes mapped to microbial metabolism in diverse environments were upregulated in response to stress (Fig. 4A). Of note, the downregulated DEGs were significantly enriched in the sulfur metabolic pathway (Fig. 4B). Sulfur is an essential component of sulfur-containing amino acids, such as cysteine and methionine, which play a crucial role in protein synthesis. The significant downregulation of sulfite reductase and sulfate/thiosulfate transporters under sodium formate stress treatment indicated that high concentrations of sodium formate could inhibit amino acid synthesis, resulting in a decrease in protein content. Furthermore, the key genes of the denitrification pathway in nitrogen metabolism, in which nitrate is ultimately reduced to nitrogen, were significantly upregulated (Fig. 4C). This was also one of the factors causing the decrease in protein content. Intriguingly, moderately weakened expression was observed in the key genes of the tetrahydrofolate cycle associated with formic acid utilization, although strain MA5 was cultured in high sodium formate medium. A complete list of the DEGs mentioned above is provided in Additional file 1: Table S6. The expression levels of some genes in the B vs. A group were further confirmed by real-time PCR (Additional file 1: Figure S3).

### Ammonium sulfate (nitrogen source) stimulates the expression of a suite of metabolic pathways associated with protein synthesis and inhibits the tetrahydrofolate cycle associated with formic acid assimilation

**Fig. 5:**
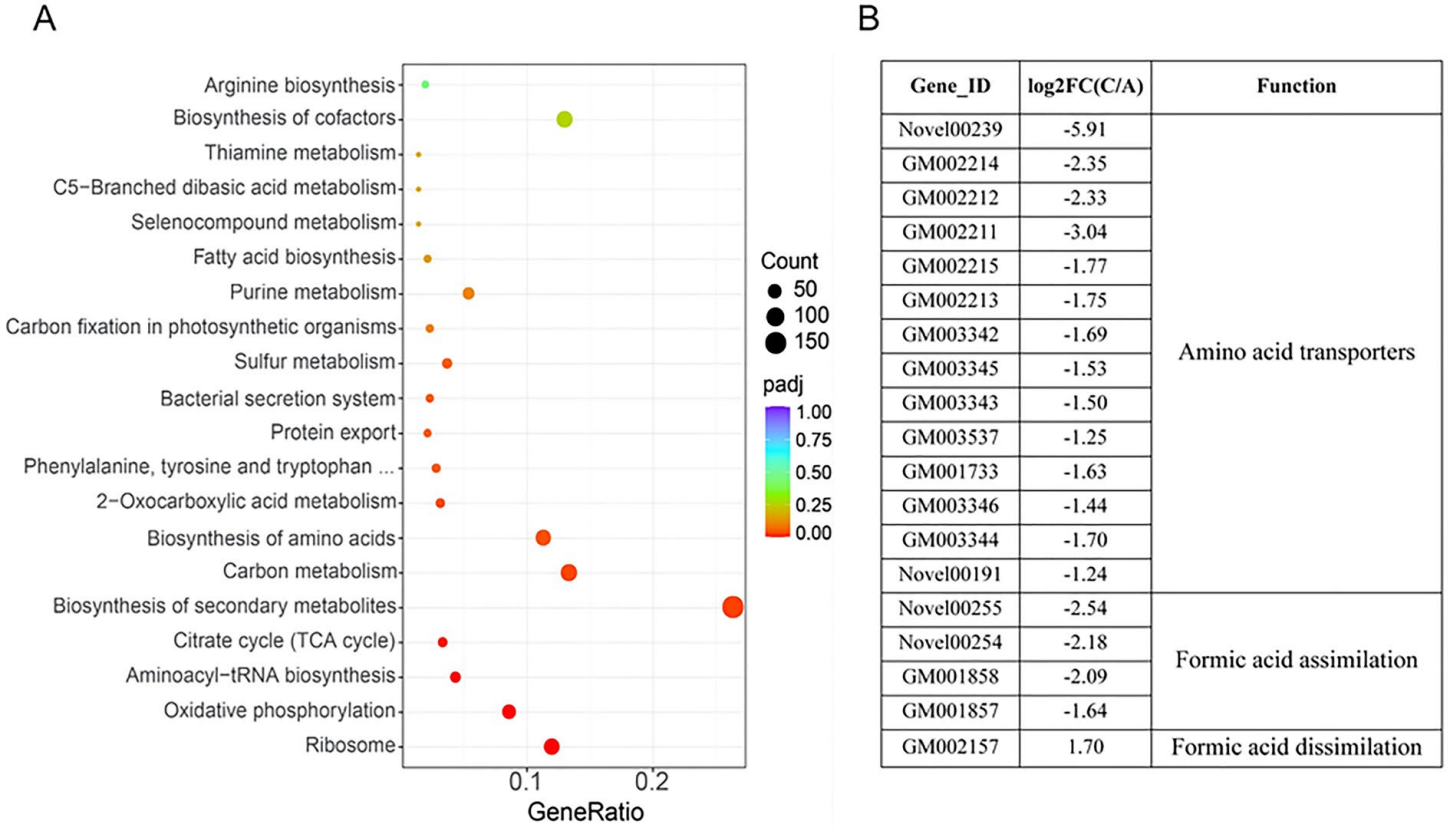
KEGG pathway enrichment (**A**) and gene information display (**B**) of DEGs in the C vs. A group. The size of the circle represents the number of DEGs in the pathway, and the color of the circle represents padj values (p value adjusted to false discovery rate), reflecting the enrichment degree

The experimental results above showed an increase in protein content and a decrease in growth rate when ammonium sulfate (replacing yeast extract) as a nitrogen source. To explain this phenomenon, the influence of ammonium sulfate on the global gene expression was analyzed via transcriptome sequencing. KEGG pathway enrichment of C vs. A group DEGs was represented in ribosome, oxidative phosphorylation, aminoacyl-tRNA biosynthesis, citrate cycle (TCA cycle), biosynthesis of amino acids, etc. (Fig. 5A). Based on the transcriptomics data, these pathways were significantly promoted by ammonium sulfate to provide more venues, vehicles, precursor substances and energy for protein synthesis. Besides, genes participating in protein export and bacterial secretion system pathways were also activated, indicating the extracellular protein content might also be increased (Fig. 5A). The DEG-related information is listed in Additional file 1: Table S7. In contrast, some amino acid transporters, such as general l-amino acid transporters, branched-chain amino acid transporters and d-methionine transporters, were significantly downregulated because ammonium sulfate is a rapidly available nitrogen source and can be directly absorbed (Fig. 5B and Additional file 1: Figure S4). The tetrahydrofolate cycle is a key pathway to formic acid metabolism. Under culture conditions with ammonium sulfate, formate-tetrahydrofolate ligase and methylene-tetrahydrofolate dehydrogenase participating in the formic acid assimilation pathway were significantly downregulated, while formate dehydrogenase catalyzing formic acid to carbon dioxide was significantly upregulated (Fig. 5B). Interestingly, the quorum sensing pathway related to cell density regulation was repressed, which might be a signal of reduced growth rate (Additional file 1: Figure S4). The expression levels of some genes in the C vs. A group were further confirmed by real-time PCR (Additional file 1: Figure S5).

## Discussion

Strain MA5 can grow in a medium containing formic acid as a single carbon source. Based on the genome sequences, a complete tetrahydrofolate cycle associated with formic acid fixation and assimilation was found (Fig. 2). In this cycle, the key enzymes dehydrogenase and hydrolase are encoded by the same gene in MA5, named *folD,* while the different coding genes corresponding to two enzymes existed in methanotrophs *Methylocystis* SB2 (Vorobev et al. 2014) and *Methylobacterium extorquens* AM1 (Crowther et al. 2008). The catalytic reaction from formic acid and tetrahydrofolate to methyltetrahydrofolate by tetrahydrofolate ligase is an irreversible reaction, and the reverse reaction is mediated by formyltetrahydrofolate deformylase. However, in *Methylobacterium extorquens* AM1, this catalytic reaction is reversible (Marx et al. 2003). The coding gene of methanol dehydrogenase catalyzing methanol to formaldehyde and the genes for the glutathione-dependent formaldehyde dehydrogenase system leading to the production of formic acid from formaldehyde were annotated in strain MA5 (Fig. 2). This methanol–formaldehyde–formic acid pathway was also reported in *Paracoccus* AK26 (Puri et al. 2020). However, the key genes encoding 3-hexulose-6-phosphate synthase and hexulose phosphate isomerase in the formaldehyde assimilation pathway were not found. This result suggests that formic acid rather than formaldehyde enters biomass synthesis metabolism when strain MA5 grows in a medium with methanol as a single carbon source.

Studies have reported that three assimilation pathways of formic acid, namely, the Calvin cycle, the RuBisCo pathway and the serine cycle, were identified in different organisms (Chistoserdova 2011; Puri et al. 2020). Except for the key genes in the serine cycle, the genes encoding enzymes for the Calvin cycle and the RuBisCo pathway were not found in the genome of strain MA5. Therefore, the serine cycle is the only pathway for the assimilation of formic acid in strain MA5, which is consistent with what has been reported in *Paracoccus* AK26 (Puri et al. 2020). Furthermore, the complete serine pathway was also found in *Methylocystis* SB2 (Vorobev et al. 2014), and both the serine cycle and the Calvin cycle are present in *Paracoccus* sp. N5 (Puri et al. 2020). Surprisingly, the gene encoding phosphoenolpyruvate carboxylase catalyzing the carboxylation of carbon dioxide and phosphoenolpyruvate into oxaloacetic acid was also annotated (Fig. 2). This discovery raises the prospect of strain MA5 and indicates that it has the ability to neutralize carbon dioxide.

In this study, comparative transcriptomic results indicated that a high concentration of sodium formate can stimulate the expression of genes responding to environmental stress, including gene enrichment in the peptidoglycan synthesis pathway and microbial metabolism in diverse environments (Fig. 4A). Studies have reported that *Lactobacillus* and *Streptococcus* can increase the proportion of monounsaturated fatty acids in the cell membrane when faced with environmental stress (Quivey et al. 2000; Montanari et al. 2010). In strain MA5, the expression of genes encoding acyl carrier protein synthetase and 3-oxygen acetyl-CoA carboxylase involved in monounsaturated fatty acid synthesis metabolism was also increased dramatically. Here, we focused on the key nodes of protein synthesis and formic acid metabolism. Based on omics analysis, an enhanced denitrification pathway and attenuated sulfur metabolism could account for the experimental result that the protein content was decreased in strain MA5 under sodium formate stress (Fig. 4B, C and Table 1). Intriguingly, formic acid consumption was decreased significantly along with moderately decreased expression levels of key genes in the tetrahydrofolate cycle under sodium formate stress treatment (Table 1 and Additional file 1: Table S6), suggesting that potential negative feedback regulation of formic acid metabolism in response to sodium formate stress exists.

Compared to yeast extract, metabolic pathways associated with protein synthesis, including ribosome, oxidative phosphorylation, aminoacyl-tRNA biosynthesis, citrate cycle (TCA cycle), and biosynthesis of amino acids, showed higher activity under ammonium sulfate culture conditions (Fig. 5A). l-Glutamate dehydrogenase, reported to catalyze ammonium and α-ketoglutaric acid to produce l-glutamic acid (Schure et al. 1995), was significantly upregulated. We believe that the increased expression of these genes is the main reason for the increased protein content in the C sample (Table 1). Notably, the significant downregulation of formic acid assimilation-relevant genes and the significant upregulation of formate dehydrogenase-encoding genes (Li et al. 2019) resulted in carbon escape from formic acid (Fig. 5B). We speculate that it is the “murderer” of reduction in biomass when strain MA5 grows under culture conditions with ammonium sulfate (Table 1). The expression levels of some representative genes detected by real-time PCR were consistent with the transcriptome profile analysis, which clearly validated the reliability of the DEGs in transcriptome analysis (Additional file 1: Figure S3, S5). Overall, our results provide a potential central metabolic network for understanding formic acid metabolism and protein synthesis in *P. communis* MA5. However, some specific metabolic and regulatory mechanisms still need to be explored to provide theoretical ground for metabolic engineering designs of this strain in the future.

## Conclusions

In conclusion, in order to detect the excellent ability to synthesize SCP directly from sodium formate, genome-scale analyses of *P. communis* MA5 were performed. Our results indicated that the complete tetrahydrofolate cycle, serine cycle, glycolytic pathway, TCA cycle and nitrogen metabolism playing key roles in the conversion of formic acid into proteins were annotated in the genome. Transcriptional analysis showed sodium formate stress could increase resistance of MA5 to environmental stress by stimulating the peptidoglycan synthesis pathway and microbial metabolism in diverse environments, but inhibit amino acid synthesis by weakening the sulfur metabolic pathway, resulting in a 31.8% decrease in protein content. Moreover, ammonium sulfate as a nitrogen source (replacing yeast extract) could increase protein content (20.4%) due to stimulation the pathways associated with protein synthesis, such as ribosome, aminoacyl-tRNA biosynthesis, TCA cycle, biosynthesis of amino acids, etc.; while decrease growth rate (62.5%) due to suppression the tetrahydrofolate cycle associated with formic acid assimilation. Our study provides theoretical guidance for the optimization of fermentation systems using formic acid as a carbon source and lays the foundation for further study on the regulatory mechanism of formic acid metabolism. In the future, genetic transformation system and gene editing system such as CRISPR–Cas9 should been established, to advance the development of *P. communis* MA5 as a SCP-based cell factory through metabolic engineering, and to elucidate the regulatory mechanism of efficient metabolism of formic acid in this strain.

### Supplementary Information

**Additional file1:** **Table S1.** Primers used in this study. **Table S2.** Concentration detection and integrity analysis of RNA. **Table S3.** Concentration detection and integrity analysis of DNA. **Table S4.** Number of genes associated with the 24 general COG functional categories. **Table S5.** Differentially expressed genes (DEGs) in both the B vs. A group and the C vs. A group. **Table S6.** Significant KEGG pathway-relevant DEGs in the B vs. A group. **Table S7.** Significant KEGG pathway-relevant DEGs in the C vs. A group. **Fig. S1** Agarose electrophoresis results of genomic DNA (A) and total RNA (B). Genomic DNA was isolated from strain MA5 grown in Group A. A1-A3: total RNA extraction from Group A; B1-B3: total RNA extraction from Group B; C1-C3: total RNA extraction from Group C. **Fig. S2 **Circular representation of the genome of strain MA5. The genome of strain MA5 consists of 3 contigs, Chr1 (1.41 MB), Chr2 (2.39 MB) and Plas1 (0.67 MB). **Fig. S3** Expression of the selected DEGs in the B vs. A group was examined by real-time PCR with *dnaN* as the reference gene. Error bars represent the standard deviation of three replicates. odc: ornithine decarboxylase; fadH: 3-oxoacyl-[acyl-carrier-protein] synthase; cysI: sulfite reductase; cdp: cell division protein; katG: peroxidase; nir: nitrite reductase. **Fig. S4** KEGG pathway enrichment of the downregulated DEGs in the C vs. A group. The size of the circle represents the number of DEGs in the pathway, and the color of the circle represents the padj value (p value adjusted to false discovery rate), reflecting the enrichment degree. **Fig. S5 **Expression of the selected DEGs in the C vs. A group was examined by real-time PCR with *dnaN* as the reference gene. Error bars represent the standard deviation of three replicates. agxT: serine transaminase; aspB: aspartate transaminase; gdh: glutamate dehydrogenase; argC: N-acetyl-gamma-glutamyl-phosphate reductase; fdh: formate dehydrogenase; argJ: glutamate N-acetyltransferase; fhs: formate-tetrahydrofolate ligase; folD: 5,10-methylene-tetrahydrofolate dehydrogenase (NADP+); aceE: pyruvate dehydrogenase E1 component; suc: 2-oxoacid dehydrogenase acyltransferase.

## Data Availability

All data are fully available without restriction.

## Abbreviations

SCP: Single-cell protein
TCA: Tricarboxylic acid
PCR: Polymerase chain reaction
DEGs: Differentially expressed genes
