# Supplementary material for: From formic acid to single-cell protein: genome-scale revealing the metabolic network of Paracoccus communis MA5
Source: Bioresour Bioprocess. 2022 May 18;9(1):55. doi: 10.1186/s40643-022-00544-0 (PMC10992362; doi:10.1186/s40643-022-00544-0)
Supplement: Supplementary file 1 — Additional file1: Table S1. Primers used in this study. Table S2. Concentration detection and integrity analysis of RNA. Table S3. Concentration detection and integrity analysis of DNA. Table S4. Number of genes associated with the 24 general COG functional categories. Table S5. Differentially expressed genes (DEGs) in both the B vs. A group and the C vs. A group. Table S6. Significant KEGG pathway-relevant DEGs in the B vs. A group. Table S7. Significant KEGG pathway-relevant DEGs in the C vs. A group. Fig. S1 Agarose electrophoresis results of genomic DNA (A) and total RNA (B). Genomic DNA was isolated from strain MA5 grown in Group A. A1-A3: total RNA extraction from Group A; B1-B3: total RNA extraction from Group B; C1-C3: total RNA extraction from Group C. Fig. S2 Circular representation of the genome of strain MA5. The genome of strain MA5 consists of 3 contigs, Chr1 (1.41 MB), Chr2 (2.39 MB) and Plas1 (0.67 MB). Fig. S3 Expression of the selected DEGs in the B vs. A group was examined by real-time PCR with dnaN as the reference gene. Error bars represent the standard deviation of three replicates. odc: ornithine decarboxylase; fadH: 3-oxoacyl-[acyl-carrier-protein] synthase; cysI: sulfite reductase; cdp: cell division protein; katG: peroxidase; nir: nitrite reductase. Fig. S4 KEGG pathway enrichment of the downregulated DEGs in the C vs. A group. The size of the circle represents the number of DEGs in the pathway, and the color of the circle represents the padj value (p value adjusted to false discovery rate), reflecting the enrichment degree. Fig. S5 Expression of the selected DEGs in the C vs. A group was examined by real-time PCR with dnaN as the reference gene. Error bars represent the standard deviation of three replicates. agxT: serine transaminase; aspB: aspartate transaminase; gdh: glutamate dehydrogenase; argC: N-acetyl-gamma-glutamyl-phosphate reductase; fdh: formate dehydrogenase; argJ: glutamate N-acetyltransferase; fhs: formate-tetrahydrofolate l [file 40643_2022_544_MOESM1_ESM.docx]

**From formic acid to single cell protein: genome-scale revealing the metabolic network of** ***Paracoccus communis* MA5**

Sheng Tong^1,3^, Lizhi Zhao^1,2^, Daling Zhu^2^, Wuxi Chen^1,3,^ Limei Chen^1,3^, Demao Li^1,3*^

^1^ Tianjin Key Laboratory for Industrial Biological Systems and Bioprocessing Engineering, Tianjin Institute of Industrial Biotechnology, Chinese Academy of Science, Tianjin 300308, China

^2^ Tianjin Key Laboratory of Brine Chemical Engineering and Resource Eco-utilization, Tianjin University of Sciences and Technology, Tianjin, 300457, China

^3^ National Innovation Centre for Synthetic Biology, Tianjin 300308, China

^*^Corresponding author:

Demao Li

86-(022)-84861993

E-mail: li_dm@tib.cas.cn

**Table S1** Primers used in this study

| Name | Forward primer (5’-3’) | Reverse primer (5’-3’) |
| --- | --- | --- |
| odc | ATCATCTTTGGCCCGACCTG | CTATTCAAGCGCGTTCACGG |
| fadH | CTGGCGCATGATTTCCAGAC | TCCATGTGAAAGCGGTCCTC |
| cysI | CCGAAGCTGGTCGATATCCC | AACTGGAACTCGGCATGGTC |
| cdp | TTCAAGATCTTCCCGGTGGC | ACGTTCGAGGATTTGGCGAT |
| katG | CTTCGTGAACCTGACCGACA | CGAGTTCGAGCCGAATACCA |
| nir | CCGAAGCTGGTCGATATCCC | AACTGGAACTCGGCATGGTC |
| agxT | TACGTGCAGCCGATCAACTT | AGATTGTCCATAGCCCGCAC |
| aspB | AAGCTGCAATCGCAATCGAC | ATAGACGTAGAACGCCCCCT |
| gdh | CATGCCGAAGATGCCGAAAG | CGTTCTTGCAGGTCATCCCT |
| argC | CTGGACGACATCATCTGCGA | GCCGAGAATTCCTGGTCGAA |
| fdh | ATATGGCGGGTTGTTCGAGG | CTCTTTCGTCGCCAGTTCCT |
| argJ | CTTCACCAAGTCCTCGACCC | TTGCCCGAGTTCACGATGAT |
| fhs | CAGTACAGCTTCACCACCGA | CCGTTCAGTCCGATCACCTC |
| folD | TGAACAACGATCCCGAGGTG | GCAGCCCGACATTCGAGATA |
| aceE | AAGCGCATCACCTCCTATCG | TAGAAATGCTTCTCGCGGCT |
| suc | CGCTTTCTCCGACCATGGAA | CTTGTCGGTCTCGATCTCGG |
| dnaN | CGGCTTCAACGCCAAATACC | GGCATGACGACATAAACCGC |

**Table S2** Concentration detection and integrity analysis of RNA

| Sample | Concentration（ng/μL） | RNA [integrity](D:/%E8%BD%AF%E4%BB%B6/Dict/8.9.6.0/resultui/html/index.html#/javascript:;) number (RIN) |
| --- | --- | --- |
| A1 | 475 | 8.20 |
| A2 | 493 | 8.00 |
| A3 | 390 | 8.40 |
| B1 | 554 | 8.20 |
| B2 | 450 | 8.20 |
| B3 | 934 | 8.10 |
| C1 | 398 | 8.00 |
| C2 | 426 | 8.00 |
| C3 | 315 | 8.00 |

**Table S3** Concentration detection and integrity analysis of DNA

| [Concentration](D:/%E8%BD%AF%E4%BB%B6/Dict/8.9.6.0/resultui/html/index.html#/javascript:;) (ng/μL) | Total quantity (μg) | The result for gel electrophoresis |
| --- | --- | --- |
| 84.2 | 23.6 | The main band is above 20K, with slight degradation |

**Table S4** Number of genes associated with the 24 general COG functional categories

| **Code** | **Number** | **Description** |
| --- | --- | --- |
| B | 1 | Chromatin structure and dynamics |
| C | 290 | Energy production and conversion |
| D | 40 | Cell cycle control, cell division, chromosome partitioning |
| E | 465 | Amino acid transport and metabolism |
| F | 103 | Nucleotide transport and metabolism |
| G | 246 | Carbohydrate transport and metabolism |
| H | 198 | Coenzyme transport and metabolism |
| I | 195 | Lipid transport and metabolism |
| J | 219 | Translation, ribosomal structure and biogenesis |
| K | 264 | Transcription |
| L | 130 | Replication, recombination and repair |
| M | 204 | Cell wall/membrane/envelope biogenesis |
| N | 36 | Cell motility |
| O | 173 | Posttranslational modification, protein turnover, chaperones |
| P | 279 | Inorganic ion transport and metabolism |
| Q | 142 | Secondary metabolites biosynthesis, transport and catabolism |
| R | 368 | General function prediction only |
| S | 238 | Function unknown |
| T | 139 | Signal transduction mechanisms |
| U | 35 | Intracellular trafficking, secretion, and vesicular transport |
| V | 85 | Defense mechanisms |
| W | 2 | Extracellular structures |
| X | 88 | Mobilome: prophages, transposons |
| Z | 1 | Cytoskeleton |

**Table S5** Differentially expressed genes (DEGs) in both the B vs. A group and the C vs. A group

| **Gene_ID** | **log2FC(B/A)** | **log2FC(C/A)** | **Gene_description** |
| --- | --- | --- | --- |
| GM001609 | -5.97 | -7.63 | Acyl-CoA dehydrogenase |
| GM001615 | -5.64 | -6.71 | NLPA lipoprotein |
| GM001614 | -5.55 | -7.32 | NLPA lipoprotein |
| GM004267 | -5.41 | -7.22 | Taurine catabolism dioxygenase TauD, TfdA family |
| sRNA00116 | -5.37 | -6.52 |  |
| GM003222 | -5.23 | -5.53 |  |
| GM001314 | -5.06 | -6.34 | NMT1/THI5 like |
| GM001610 | -4.75 | -7.08 | Acyl-CoA dehydrogenase |
| Novel00147 | -4.68 | -5.53 |  |
| sRNA00152 | -4.67 | -8.91 |  |
| GM003849 | -4.63 | -8.88 |  |
| GM001616 | -4.59 | -7.36 | Luciferase-like monooxygenase |
| GM001611 | -4.57 | -7.16 | Luciferase-like monooxygenase |
| GM001315 | -4.53 | -6.01 | Binding-protein-dependent transport system inner membrane component |
| GM004268 | -4.50 | -7.89 | Luciferase-like monooxygenase |
| Novel00239 | -4.37 | -5.91 | NLPA lipoprotein |
| GM002971 | -4.28 | -5.50 | Bacterial regulatory helix-turn-helix protein, lysR family |
| Novel00186 | -4.23 | -8.07 |  |
| GM001474 | -4.18 | -6.18 | NADPH-dependent FMN reductase |
| GM001317 | -4.15 | -6.19 | Luciferase-like monooxygenase |
| GM000423 | -4.12 | -6.18 |  |
| GM001316 | -4.11 | -6.25 | ABC transporter |
| GM001475 | -4.02 | -6.64 | Luciferase-like monooxygenase |
| Novel00237 | -3.96 | -5.49 | Acyl-CoA dehydrogenase |
| Novel00128 | -3.88 | -5.10 | LysR substrate binding domain |
| GM001481 | -3.84 | -6.29 |  |
| GM001617 | -3.84 | -7.56 | FAD-NAD(P)-binding |
| GM001613 | -3.78 | -5.81 | ABC transporter |
| Novel00015 | -3.73 | -5.79 |  |
| Novel00240 | -3.61 | -6.13 | Luciferase-like monooxygenase |
| GM004269 | -3.61 | -7.52 | Binding-protein-dependent transport system inner membrane component |
| GM000424 | -3.60 | -6.06 |  |
| Novel00238 | -3.50 | -5.05 | Luciferase-like monooxygenase |
| Novel00225 | -3.48 | -5.77 | Taurine catabolism dioxygenase TauD, TfdA family |
| GM004198 | -3.44 | -6.44 |  |
| GM004270 | -3.34 | -7.73 | NMT1/THI5 like |
| GM000538 | -3.33 | -6.77 | Bacterial extracellular solute-binding protein |
| GM004197 | -3.29 | -5.88 |  |
| GM001618 | -3.20 | -7.22 | NADPH-dependent FMN reductase |
| sRNA00203 | -3.17 | -4.19 |  |
| GM001612 | -3.14 | -4.83 | Binding-protein-dependent transport system inner membrane component |
| Novel00082 | -3.08 | -4.54 |  |
| GM003796 | -3.04 | -7.87 | homogentisate 1,2-dioxygenase |
| GM003323 | -3.01 | -2.93 | Fructose-1-6-bisphosphatase |
| Novel00083 | -3.00 | -4.59 | Luciferase-like monooxygenase |
| Novel00023 | -2.96 | -6.28 | Binding-protein-dependent transport system inner membrane component |
| GM001480 | -2.88 | -5.86 |  |
| Novel00227 | -2.87 | -4.44 | Luciferase-like monooxygenase |
| GM004199 | -2.79 | -1.84 | Substrate binding domain of ABC-type glycine betaine transport system |
| Novel00180 | -2.78 | -6.52 | homogentisate 1,2-dioxygenase |
| GM004271 | -2.76 | -6.93 | ABC transporter |
| GM001476 | -2.71 | -6.87 | Luciferase-like monooxygenase |
| Novel00154 | -2.69 | -2.99 | Fructose-1-6-bisphosphatase |
| sRNA00103 | -2.67 | -3.85 |  |
| sRNA00149 | -2.64 | -7.04 |  |
| sRNA00187 | -2.53 | -2.57 |  |
| Novel00155 | -2.52 | -2.91 | Phosphoribulokinase / Uridine kinase family |
| Novel00232 | -2.49 | -4.96 |  |
| GM001477 | -2.48 | -6.40 |  |
| GM003324 | -2.44 | -2.35 | Phosphoribulokinase / Uridine kinase family |
| Novel00241 | -2.42 | -4.89 |  |
| Novel00229 | -2.40 | -5.10 | Luciferase-like monooxygenase |
| Novel00242 | -2.23 | -5.43 |  |
| GM001318 | -2.23 | -3.87 | Major Facilitator Superfamily |
| GM000539 | -2.13 | -7.22 | Binding-protein-dependent transport system inner membrane component |
| GM001319 | -2.06 | -3.55 | Aldo/keto reductase family |
| GM001320 | -2.03 | -3.36 | Nitroreductase family |
| GM001478 | -1.93 | -6.68 | 3,4-dihydroxy-2-butanone 4-phosphate synthase |
| Novel00230 | -1.90 | -5.21 | 3,4-dihydroxy-2-butanone 4-phosphate synthase |
| GM003074 | -1.83 | -1.83 | Acyl-CoA dehydrogenase |
| GM002210 | -1.82 | -2.89 | AMP-binding enzyme |
| sRNA00193 | -1.78 | -3.58 |  |
| GM001625 | -1.73 | -5.89 |  |
| GM003467 | -1.71 | 1.68 |  |
| GM003922 | -1.66 | -1.13 | HpcH/HpaI aldolase/citrate lyase family |
| GM001145 | -1.65 | 2.03 | Redoxin |
| GM001622 | -1.64 | -5.04 | SnoaL-like domain |
| GM001482 | -1.63 | -3.49 | Lrp/AsnC ligand binding domain |
| Novel00231 | -1.62 | -4.55 |  |
| GM000593 | -1.57 | -1.62 |  |
| GM003350 | -1.50 | 5.87 | Dehydrogenase E1 component |
| GM001619 | -1.49 | -3.45 |  |
| GM001479 | -1.45 | -5.62 | FAD-NAD(P)-binding |
| GM002190 | -1.41 | -1.39 |  |
| GM001716 | -1.33 | -1.89 |  |
| GM002644 | -1.22 | 2.10 | Transglycosylase SLT domain |
| GM003351 | -1.21 | 5.33 | Transketolase |
| GM004048 | -1.17 | 2.41 | Nitrite and sulphite reductase |
| GM002553 | -1.14 | 3.18 | Dihydroprymidine dehydrogenase domain II, 4Fe-4S cluster\|PF07992:Pyridine nucleotide-disulphide oxidoreductase |
| GM000569 | -1.12 | 1.38 | NLPA lipoprotein |
| GM001623 | -1.08 | -3.76 | Phosphoribosyl-AMP cyclohydrolase |
| GM003353 | -1.08 | 4.63 | Enoyl-(Acyl carrier protein) reductase |
| GM003352 | -1.07 | 5.07 | alpha/beta hydrolase fold\|PF00364:Biotin-requiring enzyme |
| GM002757 | -1.00 | 1.33 | AMP-binding enzyme |
| GM003354 | -0.96 | 4.71 | Carboxymuconolactone decarboxylase family |
| GM003355 | -0.85 | 4.17 | Zinc-binding dehydrogenase |
| GM003405 | -0.75 | -1.01 |  |
| GM000544 | -0.70 | -1.53 |  |
| GM004241 | -0.66 | 2.74 | Methylenetetrahydrofolate reductase |
| GM003500 | -0.62 | 1.67 | DNA / pantothenate metabolism flavoprotein |
| GM004140 | 0.84 | -1.14 | Metallo-beta-lactamase superfamily |
| GM003726 | 0.90 | 2.01 | DNA methylase |
| GM002158 | 0.91 | 2.01 | Molybdopterin oxidoreductase\|PF01568:Molydopterin dinucleotide binding domain |
| GM001370 | 0.96 | 1.24 | Mur ligase family |
| GM002679 | 1.02 | 2.21 | Penicillin-binding Protein dimerisation domain |
| GM004361 | 1.03 | 1.26 | Aminotransferase class I and II |
| GM000984 | 1.06 | 1.72 | Amino acid kinase family |
| GM003674 | 1.11 | 4.70 | EVE domain |
| GM001908 | 1.12 | 1.29 | ABC transporter |
| GM001365 | 1.21 | 1.49 | D-ala D-ala ligase C-terminus\|PF01820:D-ala D-ala ligase N-terminus |
| GM001885 | 1.23 | 1.50 | 3-Oxoacyl-[acyl-carrier-protein (ACP)] synthase III |
| GM001662 | 1.27 | -1.75 |  |
| GM001233 | 1.28 | 2.05 | Glycosyl hydrolase family |
| GM002678 | 1.32 | 2.29 |  |
| GM000761 | 1.38 | -1.30 | Tripartite ATP-independent periplasmic transporter |
| Novel00109 | 1.48 | -1.45 | ABC transporter |
| GM004088 | 1.49 | 3.12 |  |
| GM000789 | 1.51 | 2.26 | Ribonucleotide reductase, small chain |
| GM001363 | 1.51 | 1.28 | Cell division protein FtsA |
| GM002868 | 1.59 | -1.71 | Putative nucleotidyltransferase |
| GM003588 | 1.66 | 4.18 | Conserved hypothetical protein 698 |
| Novel00033 | 1.69 | 2.32 |  |
| sRNA00145 | 1.74 | 1.99 |  |
| sRNA00022 | 1.75 | -2.66 |  |
| GM002869 | 1.75 | -2.01 |  |
| GM000068 | 1.77 | 1.44 | Radical SAM superfamily |
| sRNA00019 | 1.77 | -3.36 |  |
| GM000762 | 1.79 | -1.57 | Tripartite ATP-independent periplasmic transporters |
| GM000763 | 1.81 | -2.16 | Bacterial extracellular solute-binding protein, family 7 |
| Novel00030 | 1.83 | 3.11 | Peptidase family U32 |
| sRNA00096 | 1.84 | 2.69 |  |
| GM001884 | 1.90 | 2.13 | Bacterial regulatory proteins, tetR family |
| GM001661 | 1.93 | -1.44 | Bacterial extracellular solute-binding proteins |
| Novel00224 | 1.94 | 2.14 | Pyridoxal-dependent decarboxylase, pyridoxal binding domain |
| GM002870 | 1.95 | -1.51 | Response regulator receiver domain |
| GM004243 | 2.01 | 2.78 | Pyridoxal-dependent decarboxylase |
| GM001372 | 2.04 | 2.32 | Cell cycle protein |
| GM000650 | 2.06 | 2.34 | SCP-2 sterol transfer family |
| GM000651 | 2.06 | 1.95 | 3-octaprenyl-4-hydroxybenzoate carboxy-lyase |
| GM001668 | 2.22 | -1.98 | Haloacid dehalogenase-like hydrolase |
| GM002676 | 2.43 | 2.66 | MraZ protein, putative antitoxin-like |
| GM001503 | 2.48 | 1.80 | CoA-transferase family III |
| Novel00235 | 2.55 | 2.00 | CoA-transferase family III |
| GM000656 | 2.56 | 3.73 |  |
| Novel00236 | 2.74 | 2.47 | Acyl-CoA dehydrogenase, middle domain |
| GM002289 | 3.02 | -2.50 |  |
| GM001504 | 3.98 | 4.10 | Acyl-CoA dehydrogenase |
| GM002535 | 5.37 | 2.83 | Cytochrome C and Quinol oxidase polypeptide I |
| GM002534 | 6.47 | 2.99 | Cytochrome c |

**Table S6** Significant KEGG pathway-relevant DEGs in the B vs. A group

| **Gene_ID** | **log2FC(B/A)** | **Gene_description** |
| --- | --- | --- |
| Peptidoglycan biosynthesis | |  |
| GM001370 | 0.96 | Mur ligase family, glutamate ligase domain |
| GM001371 | 0.92 | Glycosyltransferase family |
| GM001365 | 1.21 | D-ala D-ala ligase |
| GM002679 | 1.02 | Penicillin binding protein transpeptidase domain |
| Microbial metabolism in diverse environments | |  |
| GM002534 | 6.47 | Cytochrome c |
| GM002531 | 5.55 | Cytochrome C oxidase, cbb3-type, subunit III |
| GM002535 | 5.37 | Cytochrome C and Quinol oxidase polypeptide I |
| Novel00112 | 4.76 | Cytochrome c |
| GM001504 | 3.98 | Acyl-CoA dehydrogenase |
| GM001668 | 2.22 | Haloacid dehalogenase-like hydrolase |
| Novel00111 | 3.58 | Cytochrome D1 heme domain |
| Novel00236 | 2.74 | Acyl-CoA dehydrogenase, middle domain |
| Novel00235 | 2.55 | CoA-transferase family III |
| GM000861 | 1.22 | Pyridoxal-dependent decarboxylase, pyridoxal binding domain |
| GM004284 | 1.07 | Biotin carboxylase, N-terminal domain |
| GM002158 | 0.91 | Molybdopterin oxidoreductase |
| GM002181 | 0.89 | C19PQQ enzyme repeat |
| GM001570 | 1.10 | Protocatechuate 3,4-dioxygenase beta subunit N terminal |
| GM002530 | 2.01 | Tetrapyrrole (Corrin/Porphyrin) Methylases |
| Sulfur metabolism | |  |
| GM001314 | -5.06 | NMT1/THI5 like |
| GM001315 | -4.53 | Binding-protein-dependent transport system inner membrane component |
| GM001316 | -4.11 | ABC transporter |
| GM001474 | -4.18 | NADPH-dependent FMN reductase |
| GM004269 | -3.61 | Binding-protein-dependent transport system inner membrane component |
| GM000538 | -3.33 | Bacterial extracellular solute-binding protein |
| GM001476 | -2.71 | Luciferase-like monooxygenase |
| GM004048 | -1.17 | Nitrite/Sulfite reductase ferredoxin-like half domain |
| GM000539 | -2.13 | Binding-protein-dependent transport system inner membrane component |
| Denitrification pathway | |  |
| GM002534 | 6.47 | Cytochrome c |
| GM002531 | 5.55 | Cytochrome C oxidase, cbb3-type, subunit III |
| GM002535 | 5.37 | Cytochrome C and Quinol oxidase polypeptide I |
| Tetrahydrofolate cycle | |  |
| GM004241 | -0.66 | Methylenetetrahydrofolate reductase |
| GM002398 | -0.95 | Tetrahydrofolate dehydrogenase/cyclohydrolase, NAD(P)-binding domain |
| Novel00255 | -0.97 | Formate-tetrahydrofolate ligase |
| GM001973 | -2.70 | Tetrahydrofolate dehydrogenase/cyclohydrolase, NAD(P)-binding domain |
| GM001858 | -0.84 | Formate-tetrahydrofolate ligase |
| Novel00254 | -0.69 | Formate-tetrahydrofolate ligase\|PF00763:Tetrahydrofolate dehydrogenase/cyclohydrolase, catalytic domain |
| GM001857 | -0.59 | Tetrahydrofolate dehydrogenase/cyclohydrolase, NAD(P)-binding domain |
| GM003190 | -0.29 | Formate-tetrahydrofolate ligase |

**Table S7** Significant KEGG pathway-relevant DEGs in the C vs. A group

| **Gene_ID** | **log2FC(C/A)** | **Gene_description** |
| --- | --- | --- |
| Ribosome | |  |
| Novel00201 | 3.87 | Ribosomal protein L4/L1 family |
| GM003962 | 4.58 | Ribosomal protein L3 |
| GM003976 | 4.59 | Ribosomal protein L10 |
| GM003961 | 4.45 | Ribosomal protein L4/L1 family |
| GM003830 | 4.71 | Ribosomal protein L13 |
| GM000957 | 4.07 | Ribosomal protein TL5 |
| GM003978 | 4.37 | Ribosomal protein L11 |
| GM003942 | 3.67 | Ribosomal proteins 50S-L15, 50S-L18e, 60S-L27A |
| GM000418 | 3.91 | Ribosomal protein S16 |
| GM003886 | 3.99 |  |
| Novel00206 | 3.63 | Ribosomal protein L7/L12 dimerisation domain |
| Novel00196 | 3.83 | Ribosomal proteins 50S L24/mitochondrial 39S L24 |
| GM003951 | 4.41 | Ribosomal proteins 50S L24/mitochondrial 39S L24 |
| Novel00202 | 3.91 | Ribosomal protein S10p/S20e |
| GM003952 | 4.36 | Ribosomal protein L14p/L23e |
| GM003945 | 4.13 | Ribosomal protein S5 |
| GM003977 | 4.12 | Ribosomal protein L1p/L10e family |
| GM003975 | 4.32 | Ribosomal protein L7/L12 |
| Novel00200 | 3.85 |  |
| GM003944 | 4.15 |  |
| Novel00208 | 3.33 | Ribosomal protein L11, RNA binding domain |
| Novel00195 | 3.62 | ribosomal L5P family |
| GM003963 | 4.56 | Ribosomal protein S10p/S20e |
| GM003950 | 4.19 | ribosomal L5P family |
| GM002591 | 3.77 |  |
| GM003959 | 3.90 | Ribosomal Proteins L2 |
| Novel00193 | 3.29 | Ribosomal protein S5 |
| GM003960 | 4.14 |  |
| GM003871 | 3.53 | Ribosomal protein L9 |
| GM003870 | 3.63 |  |
| GM003829 | 3.01 | Ribosomal protein S9/S16 |
| GM003954 | 4.27 |  |
| GM002218 | 3.84 |  |
| Novel00194 | 3.09 |  |
| GM003947 | 3.82 | Ribosomal protein L6 |
| GM003949 | 3.94 |  |
| GM003948 | 3.74 | Ribosomal protein S8 |
| GM003953 | 4.22 |  |
| GM003869 | 3.46 | Ribosomal protein S6 |
| GM001246 | 3.09 |  |
| GM003968 | 3.72 | Ribosomal protein S7p/S5e |
| GM003956 | 3.45 | Ribosomal protein S3 |
| Novel00197 | 3.75 | Ribosomal protein S17 |
| GM003946 | 3.65 | Ribosomal L18 |
| GM000972 | 3.85 | Ribosomal prokaryotic L21 protein |
| Novel00199 | 2.86 | Ribosomal Proteins L2 |
| GM003958 | 3.82 |  |
| GM003957 | 3.70 | Ribosomal protein L22p/L17e |
| GM001223 | 3.62 |  |
| GM003969 | 3.70 | Ribosomal protein S12/S23 |
| Novel00086 | 2.70 | Ribosomal protein S20 |
| GM001391 | 3.26 |  |
| Novel00207 | 2.91 | Ribosomal protein L1p/L10e family |
| GM003938 | 3.23 | Ribosomal protein S11 |
| GM003939 | 3.33 | Ribosomal protein S13/S18 |
| Novel00192 | 2.78 | Ribosomal protein S13/S18 |
| GM003955 | 2.93 | Ribosomal protein L16p/L10e |
| Novel00205 | 3.21 | Ribosomal protein S12/S23 |
| GM000421 | 3.13 | Ribosomal protein L19 |
| Novel00198 | 3.10 | Ribosomal protein L22p/L17e |
| GM004100 | 2.75 | Ribosomal protein L20 |
| GM000574 | 2.72 | Ribosomal protein S4/S9 |
| Novel00049 | 2.73 | Ribosomal prokaryotic L21 protein |
| GM000422 | 2.93 |  |
| GM003936 | 2.77 | Ribosomal protein L17 |
| GM004101 | 2.76 |  |
| Novel00190 | 3.46 |  |
| GM000971 | 2.67 |  |
| GM003604 | 1.93 | Ribosomal protein S2 |
| GM003600 | 2.03 | S1 RNA binding domain |
| Oxidative phosphorylation | |  |
| GM001046 | 3.93 | DHH family |
| GM002661 | 4.28 | Succinate dehydrogenase/Fumarate reductase transmembrane subunit |
| GM002660 | 3.99 | Succinate dehydrogenase/Fumarate reductase transmembrane subunit |
| GM002662 | 3.63 | FAD binding domain, Fumarate reductase flavoprotein C-term |
| GM003100 | 3.30 | ATP synthase delta (OSCP) subunit |
| GM003101 | 3.48 | ATP synthase alpha/beta family |
| GM004220 | 3.56 | Ubiquitinol-cytochrome C reductase Fe-S subunit TAT signal |
| GM003035 | 3.14 | ATP synthase A chain |
| GM003102 | 2.78 | ATP synthase |
| GM002731 | 2.77 | Respiratory-chain NADH dehydrogenase, 49 Kd subunit |
| GM004221 | 3.26 | Cytochrome b/b6/petB |
| GM003103 | 2.90 | ATP synthase alpha/beta family |
| GM002729 | 3.01 | Respiratory-chain NADH dehydrogenase, 30 Kd subunit |
| GM002727 | 3.20 | NADH-ubiquinone/plastoquinone oxidoreductase, chain 3 |
| GM000559 | 2.48 | Cytochrome c oxidase assembly protein CtaG/Cox11 |
| GM001198 | 2.77 | Polyphosphate kinase 2 (PPK2) |
| GM002732 | 2.46 | Thioredoxin-like [2Fe-2S] ferredoxin |
| GM004222 | 2.67 | Cytochrome C1 family |
| GM002728 | 2.56 | NADH ubiquinone oxidoreductase, 20 Kd subunit |
| GM003104 | 2.44 | ATP synthase, Delta/Epsilon chain, beta-sandwich domain |
| GM000556 | 2.74 | Cytochrome C oxidase subunit II, transmembrane domain |
| GM002735 | 2.13 | Respiratory-chain NADH dehydrogenase 51 Kd subunit |
| GM000834 | 2.16 | Polyphosphate kinase 2 (PPK2) |
| GM001212 | 2.78 | Cytochrome C and Quinol oxidase polypeptide I |
| GM000995 | 1.94 | Cytochrome oxidase assembly protein |
| GM003077 | 2.73 | Cytochrome c oxidase subunit III |
| GM000557 | 2.04 | UbiA prenyltransferase family |
| GM003037 | 2.07 | ATP synthase B/B' CF(0) |
| GM003036 | 2.27 |  |
| GM002747 | 2.00 | NADH-ubiquinone/plastoquinone oxidoreductase chain 6 |
| GM002748 | 2.44 | NADH-ubiquinone/plastoquinone oxidoreductase chain 4L |
| GM002744 | 1.74 | 4Fe-4S dicluster domain |
| GM000849 | 1.54 | Polyphosphate kinase middle domain |
| GM003076 | 2.18 | Prokaryotic Cytochrome C oxidase subunit IV |
| GM001838 | 1.76 | Cytochrome bd terminal oxidase subunit I |
| GM002742 | 2.01 | NADH dehydrogenase |
| GM002749 | 1.60 | Proton-conducting membrane transporter |
| GM003078 | 2.32 | Cytochrome C and Quinol oxidase polypeptide I |
| GM001211 | 2.21 | Cytochrome C oxidase, mono-heme subunit/FixO |
| GM000560 | 1.94 | Cytochrome c oxidase subunit III |
| GM000483 | 1.87 | Cytochrome C and Quinol oxidase polypeptide I |
| GM003038 | 1.66 | ATP synthase B/B' CF(0) |
| GM002750 | 1.50 | Proton-conducting membrane transporter |
| GM002740 | 1.41 | NADH-ubiquinone oxidoreductase-G iron-sulfur binding region |
| GM001210 | 1.97 |  |
| GM003379 | -1.25 | Cytochrome C and Quinol oxidase polypeptide I |
| GM002665 | 1.58 | 4Fe-4S dicluster domain |
| GM002751 | 1.18 | Proton-conducting membrane transporter |
| GM001637 | 1.69 | Protein of unknown function (DUF1007) |
| GM003079 | 2.00 | COX Aromatic Rich Motif |
| Aminoacyl-tRNA biosynthesis | |  |
| GM000490 | 3.57 | GatB/GatE catalytic domain |
| GM002692 | 3.46 | tRNA synthetases class II (A) |
| GM002093 | 3.25 | tRNA synthetases class I (M) |
| GM002800 | 3.17 | tRNA synthetases class II (D, K and N) |
| GM000505 | 2.91 | tRNA synthetases class I (W and Y) |
| GM002631 | 3.14 | Glycyl-tRNA synthetase alpha subunit |
| GM004093 | 2.87 | tRNA synthetase B5 domain |
| GM000852 | 2.81 | tRNA synthetase class II core domain (G, H, P, S and T) |
| GM003874 | 2.79 | tRNA synthetases class I (E and Q), catalytic domain |
| GM003509 | 2.76 | tRNA synthetases class I (W and Y) |
| GM000942 | 2.70 | tRNA synthetases class I (C) catalytic domain |
| GM004254 | 2.61 | tRNA synthetases class I (I, L, M and V) |
| GM003471 | 2.54 | tRNA synthetase class II core domain (G, H, P, S and T) |
| GM001097 | 2.37 | tRNA synthetases class I (I, L, M and V) |
| GM002669 | 2.48 |  |
| GM001235 | 2.29 | Arginyl tRNA synthetase N terminal domain |
| GM003717 | 2.50 | tRNA synthetases class I (M) |
| GM003194 | 2.38 | tRNA synthetases class I (K) |
| GM002633 | 2.03 | Glycyl-tRNA synthetase beta subunit |
| GM002587 | 2.17 | tRNA synthetase class II core domain (G, H, P, S and T) |
| GM000493 | 2.06 | Histidyl-tRNA synthetase |
| GM004096 | 2.19 | Aminoacyl tRNA synthetase class II, N-terminal domain |
| GM002670 | 1.92 | Amidase |
| GM000826 | 1.20 | tRNA synthetases class I (E and Q), catalytic domain |
| GM004320 | -0.87 | L-seryl-tRNA selenium transferase |
| Citrate cycle (TCA cycle) | |  |
| GM002713 | 5.03 | Pyridine nucleotide-disulphide oxidoreductase, dimerisation domain |
| GM000431 | 4.60 | Dehydrogenase E1 component |
| GM000430 | 3.87 | Biotin-requiring enzyme, Transketolase, pyrimidine binding domain |
| GM002650 | 3.95 | 2-oxoglutarate dehydrogenase C-terminal |
| GM003838 | 3.87 | Isocitrate/isopropylmalate dehydrogenase |
| GM002661 | 4.28 | Succinate dehydrogenase/Fumarate reductase transmembrane subunit |
| GM000509 | 3.85 | Fumarase C C-terminus |
| GM002660 | 3.99 | Succinate dehydrogenase/Fumarate reductase transmembrane subunit |
| GM002649 | 3.54 | 2-oxoacid dehydrogenases acyltransferase (catalytic domain) |
| GM002662 | 3.63 | FAD binding domain, Fumarate reductase flavoprotein C-term |
| GM000429 | 3.19 | 2-oxoacid dehydrogenases acyltransferase (catalytic domain) |
| GM002654 | 3.30 | lactate/malate dehydrogenase, alpha/beta C-terminal domain |
| GM000576 | 3.24 | Aconitase family (aconitate hydratase) |
| GM000827 | 3.14 | Citrate synthase, C-terminal domain |
| GM001852 | -2.73 | CoA-ligase |
| GM001853 | -2.48 | CoA-ligase |
| GM000568 | 2.42 | Acetyl-CoA hydrolase/transferase N-terminal domain |
| GM002645 | 1.61 | Pyridine nucleotide-disulphide oxidoreductase |
| GM002665 | 1.58 | 2Fe-2S iron-sulfur cluster binding domain |
| Biosynthesis of amino acids | |  |
| GM000939 | 5.00 | Enolase, C-terminal TIM barrel domain |
| GM003838 | 3.87 | Isocitrate/isopropylmalate dehydrogenase |
| GM002492 | 3.83 | Aconitase C-terminal domain |
| GM002494 | 3.67 | Aconitase family (aconitate hydratase) |
| GM002562 | 3.40 | Arginosuccinate synthase |
| GM000876 | 3.19 | Transketolase, thiamine diphosphate binding domain |
| GM002518 | 3.91 | Transaldolase/Fructose-6-phosphate aldolase |
| GM001340 | 3.69 | Glyceraldehyde 3-phosphate dehydrogenase, NAD binding domain |
| GM002501 | 3.27 | Tetrahydrodipicolinate N-succinyltransferase N-terminal |
| GM000576 | 3.24 | Aconitase family (aconitate hydratase) |
| GM002863 | 2.89 | Amino acid kinase family |
| GM001037 | 3.23 | Ribose 5-phosphate isomerase A (phosphoriboisomerase A) |
| GM002411 | 3.25 | Amino-transferase class IV |
| Novel00084 | 2.91 | Glyceraldehyde 3-phosphate dehydrogenase |
| GM000486 | 2.89 | Vitamin B12 dependent methionine synthase, activation domain |
| GM000498 | 3.11 | DeoC/LacD family aldolase |
| GM000827 | 3.14 | Citrate synthase, C-terminal domain |
| GM003280 | 2.60 | Semialdehyde dehydrogenase, NAD binding domain |
| GM004273 | 2.46 | Tryptophan synthase alpha chain |
| GM000562 | 2.37 | Pyridoxal-phosphate dependent enzyme |
| GM003878 | 2.44 | Amino acid kinase family |
| GM001238 | 2.41 | S-adenosylmethionine synthetase, central domain |
| GM003245 | 2.54 | Aminotransferase class I and II |
| GM003546 | 3.72 | Pyridoxal-phosphate dependent enzyme |
| GM000535 | 2.30 | Imidazoleglycerol-phosphate dehydratase |
| GM003695 | 2.10 | Argininosuccinate lyase C-terminal |
| GM002756 | 2.05 | EPSP synthase (3-phosphoshikimate 1-carboxyvinyltransferase) |
| GM003108 | 2.27 | N-terminal domain of ribose phosphate pyrophosphokinase |
| GM000702 | 2.17 | Aminotransferase class I and II |
| GM003595 | 2.24 | Pyridoxal-phosphate dependent enzyme |
| GM004231 | -2.50 |  |
| GM000500 | 1.98 | Phosphoglycerate kinase |
| GM004178 | 2.03 | Diaminopimelate epimerase |
| GM002920 | 1.94 | Isocitrate/isopropylmalate dehydrogenase |
| GM002853 | 1.74 | Anthranilate synthase component I, N terminal region\| |
| GM004074 | 1.75 | Aminotransferase class I and II |
| GM002849 | 2.01 | Glutamine amidotransferase class-I |
| GM002441 | 2.01 | Ribulose-phosphate 3 epimerase family |
| GM000399 | 2.00 | Pyridoxal-phosphate dependent enzyme |
| GM000050 | 1.74 | Dehydratase family |
| GM000457 | 1.77 | Histidinol dehydrogenase |
| GM001623 | -3.76 | Phosphoribosyl-AMP cyclohydrolase |
| GM003713 | 1.82 | Aminotransferase class-III |
| GM000545 | 1.56 | Chorismate synthase |
| GM004103 | 1.79 | Pyruvate kinase, barrel domain |
| GM003531 | 1.86 | Class-II DAHP synthetase family |
| GM000572 | 1.41 | Triosephosphate isomerase |
| GM000417 | 2.02 |  |
| GM002813 | 1.45 | Small subunit of acetolactate synthase |
| GM002615 | 1.42 | Ornithine cyclodeaminase/mu-crystallin family |
| GM002207 | 1.74 | Dehydroquinase class II |
| GM001173 | 1.32 | Prephenate dehydratase |
| GM003657 | 1.41 | Dehydratase family |
| GM003597 | 1.39 | N-(5'phosphoribosyl)anthranilate (PRA) isomerase |
| GM004055 | 1.29 | Aminotransferase class I and II |
| GM003711 | 1.67 | Aspartate/ornithine carbamoyltransferase, carbamoyl-P binding domain |
| GM000911 | 1.57 | LeuA allosteric (dimerisation) domain |
| GM000532 | 1.40 | Histidine biosynthesis protein |
| GM002416 | 1.21 | ArgJ family |
| GM001511 | 1.37 | DAHP synthetase I family |
| GM002127 | 1.17 | Dihydrodipicolinate reductase |
| GM002142 | 1.20 | Shikimate / quinate 5-dehydrogenase |
| GM003548 | 1.52 | Ornithine cyclodeaminase/mu-crystallin family |
| GM001313 | 1.48 | Glutamine synthetase, beta-Grasp |
| GM003510 | 1.21 | Homocysteine S-methyltransferase |
| GM001379 | 1.53 | Aldehyde dehydrogenase family |
| Protein export | |  |
| GM000415 | 3.49 | Signal peptide binding domain, SRP54-type protein, GTPase domain |
| GM003881 | 3.08 | 60Kd inner membrane protein\|PF14849:YidC periplasmic domain |
| GM004153 | 2.89 | Peptidase S24-like |
| GM003980 | 2.55 |  |
| GM000831 | 2.39 | Preprotein translocase SecG subunit |
| GM002113 | 2.33 | Preprotein translocase subunit SecB |
| GM003237 | 1.65 | Protein export membrane protein |
| GM003238 | 1.86 | Protein export membrane protein\|PF07549:SecD/SecF GG Motif |
| GM003941 | 1.88 | SecY translocase |
| GM002414 | 1.35 | SecA DEAD-like domain |
| GM000497 | 1.14 | SRP54-type protein, GTPase domain |
| GM003236 | 1.40 | Preprotein translocase subunit |
| Bacterial secretion system | |  |
| GM000415 | 3.49 | Signal peptide binding domain, SRP54-type protein, GTPase domain |
| GM003881 | 3.08 | 60Kd inner membrane protein\|PF14849:YidC periplasmic domain |
| GM003980 | 2.55 |  |
| GM000831 | 2.39 | Preprotein translocase SecG subunit |
| GM002053 | 1.85 | Outer membrane efflux protein |
| GM002113 | 2.33 | Preprotein translocase subunit SecB |
| Novel00090 | 2.18 |  |
| GM003237 | 1.65 | Protein export membrane protein |
| GM003238 | 1.86 | Protein export membrane protein\|PF07549:SecD/SecF GG Motif |
| GM003941 | 1.88 | SecY translocase |
| GM002414 | 1.35 | SecA DEAD-like domain |
| GM000497 | 1.14 | SRP54-type protein, GTPase domain |
| GM003236 | 1.40 | Preprotein translocase subunit |

**Fig. S1**


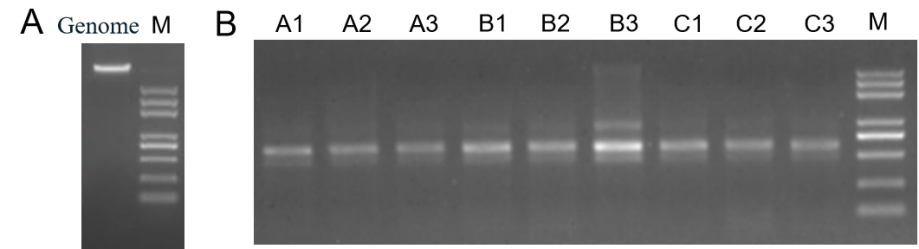


**Fig. S1** Agarose electrophoresis results of genomic DNA (A) and total RNA (B). Genomic DNA was isolated from strain MA5 grown in Group A. A1-A3: total RNA extraction from Group A; B1-B3: total RNA extraction from Group B; C1-C3: total RNA extraction from Group C.

**Fig. S2**


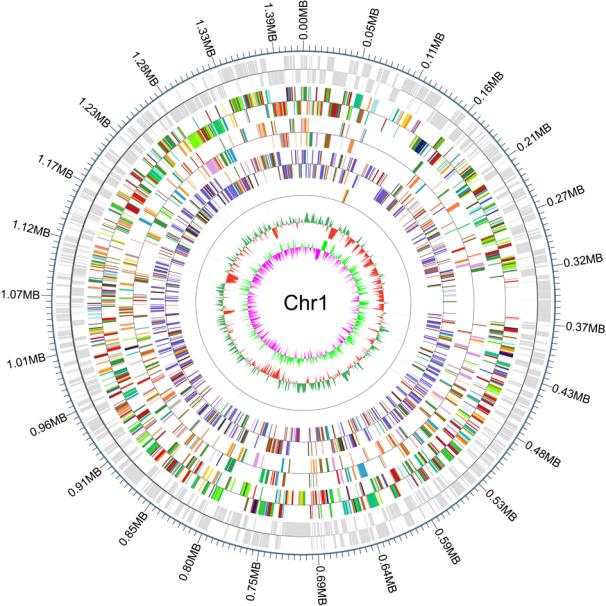

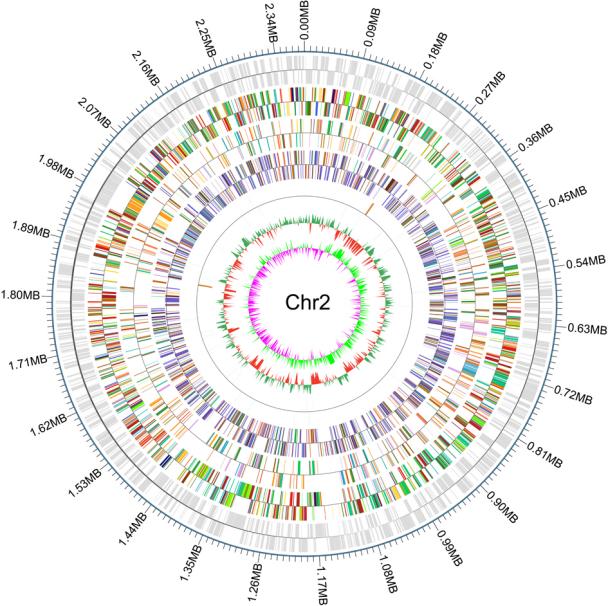

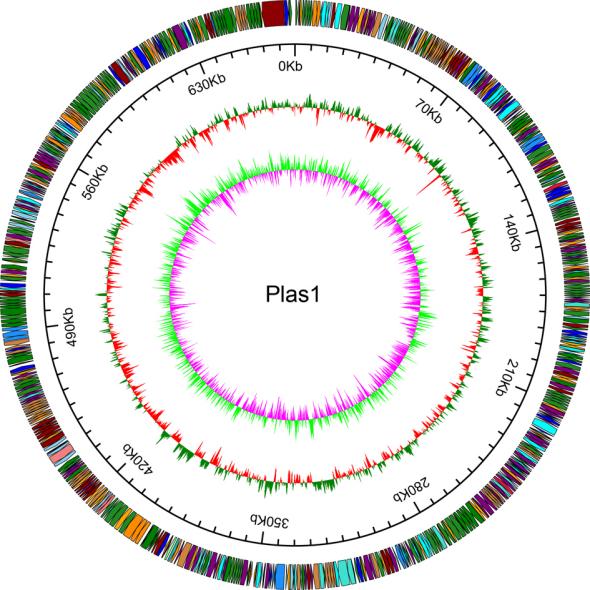


**Fig. S2** Circular representation of the genome of strain MA5. The genome of strain MA5 consists of 3 contigs, Chr1 (1.41 MB), Chr2 (2.39 MB) and Plas1 (0.67 MB).

**Fig. S3**


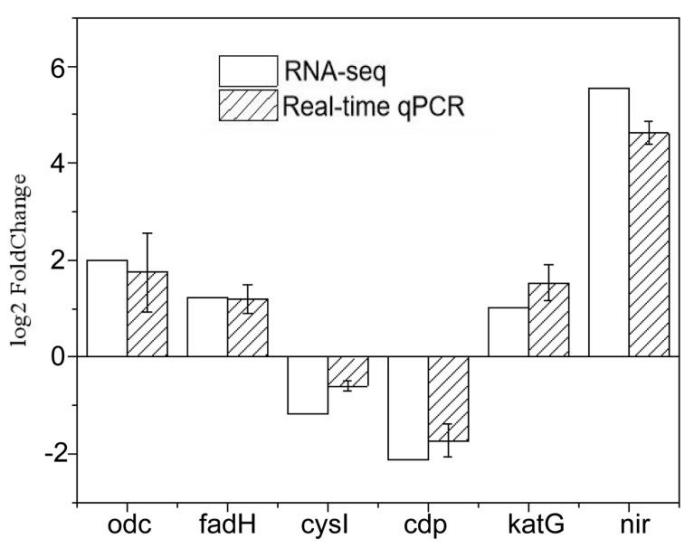


**Fig. S3** Expression of the selected DEGs in the B vs. A group was examined by real-time PCR with *dnaN* as the reference gene. Error bars represent the standard deviation of three replicates. odc: ornithine decarboxylase; fadH: 3-oxoacyl-[acyl-carrier-protein] synthase; cysI: sulfite reductase; cdp: cell division protein; katG: peroxidase; nir: nitrite reductase.

**Fig. S4**


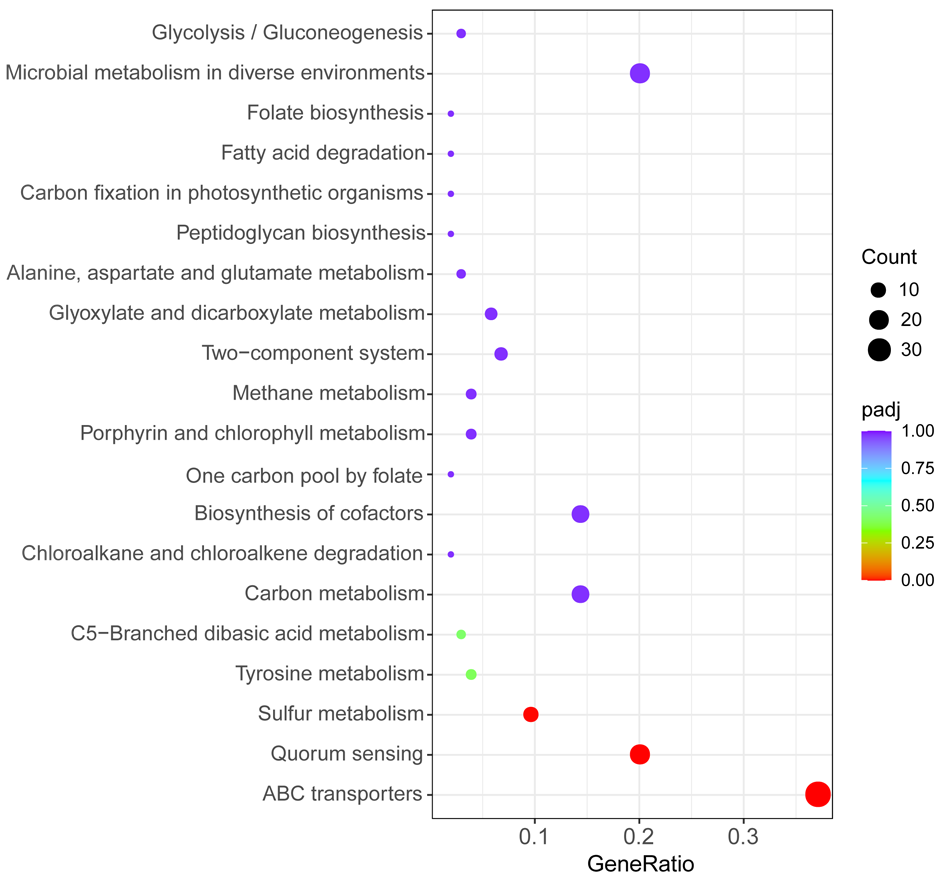


**Fig. S4** KEGG pathway enrichment of the downregulated DEGs in the C vs. A group. The size of the circle represents the number of DEGs in the pathway, and the color of the circle represents the padj value (p value adjusted to false discovery rate), reflecting the enrichment degree.

**Fig. S5**


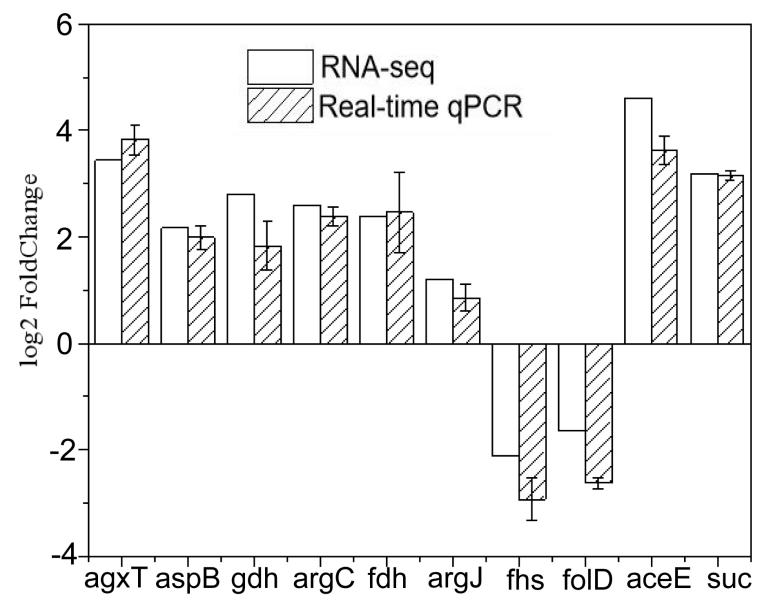


**Fig. S5** Expression of the selected DEGs in the C vs. A group was examined by real-time PCR with *dnaN* as the reference gene. Error bars represent the standard deviation of three replicates. agxT: serine transaminase; aspB: aspartate transaminase; gdh: glutamate dehydrogenase; argC: N-acetyl-gamma-glutamyl-phosphate reductase; fdh: formate dehydrogenase; argJ: glutamate N-acetyltransferase; fhs: formate-tetrahydrofolate ligase; folD: 5,10-methylenetetrahydrofolate dehydrogenase (NADP+); aceE: pyruvate dehydrogenase E1 component; suc: 2-oxoacid dehydrogenase acyltransferase.
